# Protocol for the development of the Wales Multimorbidity e-Cohort (WMC): data sources and methods to construct a population-based research platform to investigate multimorbidity

**DOI:** 10.1136/bmjopen-2020-047101

**Published:** 2021-01-19

**Authors:** Jane Lyons, Ashley Akbari, Utkarsh Agrawal, Gill Harper, Amaya Azcoaga-Lorenzo, Rowena Bailey, James Rafferty, Alan Watkins, Richard Fry, Colin McCowan, Carol Dezateux, John P Robson, Niels Peek, Chris Holmes, Spiros Denaxas, Rhiannon Owen, Keith R Abrams, Ann John, Dermot O'Reilly, Sylvia Richardson, Marlous Hall, Chris P Gale, Jan Davies, Chris Davies, Lynsey Cross, John Gallacher, James Chess, Anthony J Brookes, Ronan A Lyons

**Affiliations:** 1 Population Data Science, Swansea University Medical School, Swansea, UK; 2 School of Medicine, University of St Andrews, St Andrews, Fife, UK; 3 Barts and The London School of Medicine and Dentistry, Queen Mary University of London, London, UK; 4 Health e-Research Centre, Institute of Population Health, University of Manchester, Manchester, UK; 5 Department of Statistics, Oxford University, Oxford, Oxfordshire, UK; 6 Institute of Health Informatics, University College London, London, London, UK; 7 Department of Health Sciences, University of Leicester, Leicester, Leicestershire, UK; 8 Epidemiology and Public Health, Queens University Belfast, Belfast, UK; 9 Department of Epidemiology and Public Health, MRC Biostatistics Unit, Cambridge, UK; 10 School of Medicine, University of Leeds, Leeds, UK; 11 Members of the public, Swansea, UK; 12 Department of Psychiatry, Oxford University, Oxford, UK; 13 Renal Unit, Swansea Bay University Health Board, Swansea, UK; 14 Department of Genetics, University of Leicester, Leicester, UK

**Keywords:** public health, epidemiology, health policy, primary care, geriatric medicine

## Abstract

**Introduction:**

Multimorbidity is widely recognised as the presence of two or more concurrent long-term conditions, yet remains a poorly understood global issue despite increasing in prevalence.

We have created the Wales Multimorbidity e-Cohort (WMC) to provide an accessible research ready data asset to further the understanding of multimorbidity. Our objectives are to create a platform to support research which would help to understand prevalence, trajectories and determinants in multimorbidity, characterise clusters that lead to highest burden on individuals and healthcare services, and evaluate and provide new multimorbidity phenotypes and algorithms to the National Health Service and research communities to support prevention, healthcare planning and the management of individuals with multimorbidity.

**Methods and analysis:**

The WMC has been created and derived from multisourced demographic, administrative and electronic health record data relating to the Welsh population in the Secure Anonymised Information Linkage (SAIL) Databank. The WMC consists of 2.9 million people alive and living in Wales on the 1 January 2000 with follow-up until 31 December 2019, Welsh residency break or death. Published comorbidity indices and phenotype code lists will be used to measure and conceptualise multimorbidity.

Study outcomes will include: (1) a description of multimorbidity using published data phenotype algorithms/ontologies, (2) investigation of the associations between baseline demographic factors and multimorbidity, (3) identification of temporal trajectories of clusters of conditions and multimorbidity and (4) investigation of multimorbidity clusters with poor outcomes such as mortality and high healthcare service utilisation.

**Ethics and dissemination:**

The SAIL Databank independent Information Governance Review Panel has approved this study (SAIL Project: 0911). Study findings will be presented to policy groups, public meetings, national and international conferences, and published in peer-reviewed journals.

**Strengths and limitations of this study**

- Creation and access to a multisourced population based, deeply phenotyped e-cohort.
- Future use of this resource removes the need for data management and cleaning of source data, accelerating research and which could also support efforts for reproducibility of results.
- Variety of individual and household level data available on demography, health status, healthcare utilisation, both primary and secondary healthcare, and mortality to support a wide range of analytical approaches to addressing scientific questions.
- Input from multiple disciplines and institutions from across all four nations of the UK to help understand, measure and address multimorbidity.
- Routine data do not capture data on some important aspects, such as quality of life.

## Introduction

Multimorbidity is defined by the UK’s Academy of Medical Sciences (AMS) and World Health Organization (WHO) as the presence of two or more concurrent long-term conditions, which is a global and growing phenomenon.^1 2^ Multimorbidity is more prevalent in older individuals and associated with high healthcare utilisation and mortality, but with large numbers of patients of all age suffering from multimorbidity.^3–6^ With an ageing population, it is estimated that two in three people in England aged 65 years or over will experience multimorbidity by 2035 and nearly one fifth will have complex multimorbidity (four or more conditions).^7^

Much of what is known about multimorbidity is based on a limited and fragmented knowledge base, largely derived from studies of older people in high-income countries or hospital populations.^1 8^ The 2018 AMS report concluded that multimorbidity is an unhelpful term implying random assortment of disease when it often refers to clusters of specific diseases. Once identified, these disease clusters can be addressed specifically through research, healthcare policy development and service delivery.^1 9^ The identification of previously unrecognised disease clusters may also provide biological and clinical insights into their aetiology, prevention and treatment. The AMS report identified specific research gaps and proposed a list of priorities (box 1). Several can be addressed through a combination of health data science, epidemiology and statistics and by exploiting the potential from creating deeply phenotyped cohorts from population and clinical data sources.

Box 1 **The Academy of Medical Sciences identified research gaps**

- The scale and nature of multimorbidity and how it is changing over time.

- Which clusters of conditions cause the biggest problems for patients.
- The causes of the most common clusters including links with sex, ethnicity, income and lifestyle.
- The best ways to prevent the patients developing multimorbidity, and whether this requires different approaches to just preventing individual conditions.
- How doctors can increase the benefits and reduce the risks of treatment for patients with multimorbidity.
- How to organise healthcare systems to deal with multimorbidity more effectively and how best to use digital technology in caring for patients.

Responding to this agenda, we created a privacy protecting total population electronic cohort—the Wales Multimorbidity e-Cohort (WMC)—as a platform to study these issues in depth, collaborating with scientists from many different institutions and disciplines, clinicians, and members of the public from across the UK to create a broader team science approach.

The objectives of this work are to understand prevalence, trajectories and determinants of multimorbidity, and identify clusters causing the greatest healthcare burden. The WMC will also contribute data on incidence, prevalence and burden to the Global Burden of Diseases (GBD) Study,^10 11^ and provide new multimorbidity phenotypes to e-cohorts with local participants, and phenotyping algorithms to many e-cohorts that use routine data.^12^

We expect that findings from these analyses will provide evidence to health policy leads in order to support prevention and the complex healthcare planning and management of multimorbid individuals. Members of the public are embedded in the research team to ensure the resource focuses on issues of concern to the public.

This paper describes the creation of the WMC and the statistical approaches that will be developed to support the diverse research objectives.

## Methods

The WMC was developed by linking multiple routinely collected population and clinical data sources on the population of Wales from 2000 to 2019. We used the privacy-protecting Secure Anonymised Information Linkage (SAIL) Databank, to contribute to the Health Data Research UK National Implementation Multimorbidity Resource (HNIMR) project and extended to 2020 for the Medical Research Council (MRC) funded Welsh Multimorbidity Machine Learning project.^13 14^ SAIL is one of the most comprehensive, privacy protecting, linked data Trusted Research Environments in the UK. SAIL uses data from many different sources and provides linkage at individual and household level.^15^ It has supported many different study designs, including large-scale community-based or clinical condition-based observational studies, disease surveillance, evaluation of natural experiments of environmental interventions, embedded trials and the Dementias Platform UK.^16–23^

### Cohort design and characteristics

**Figure 1:**
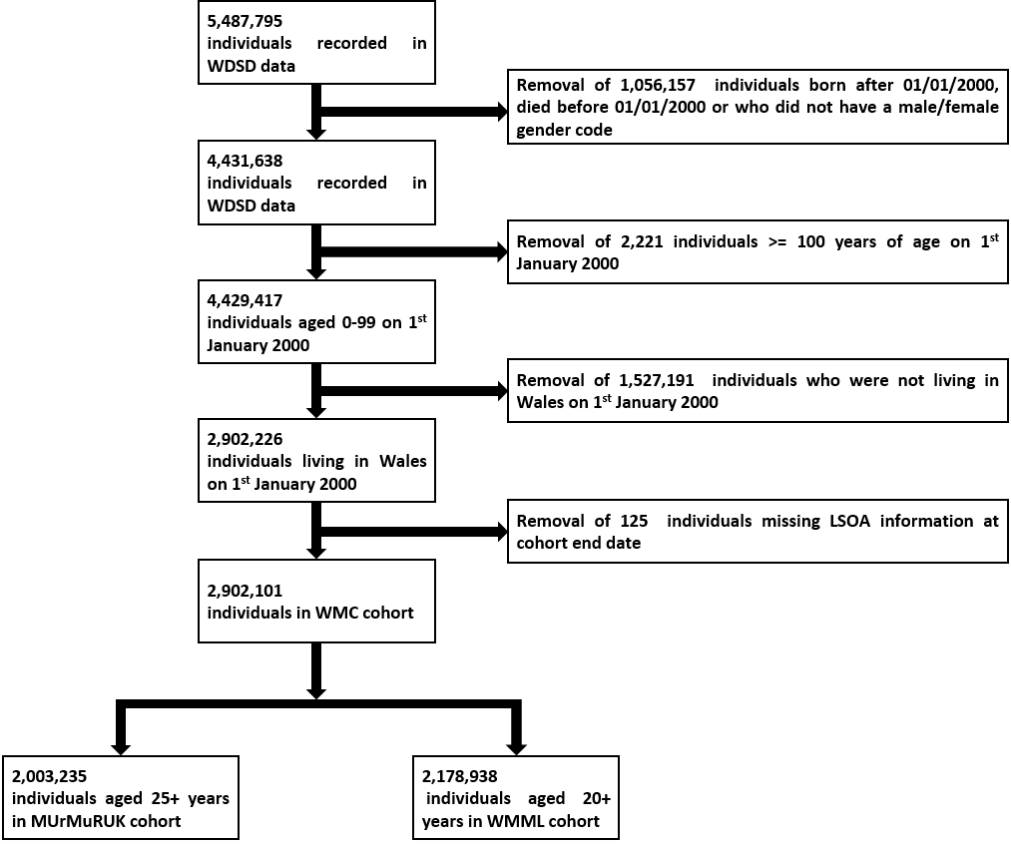
WMC flow diagram, based on inclusion criteria. LSOA; lower layer super output area, WDSD; Welsh Demographic Service Dataset, WMC; Wales Multimorbidity e-Cohort, WMML; Welsh Multimorbidity Machine Learning.

The WMC is a clearly defined complete population cohort. Cohort entry includes all residents in Wales, alive and living on 1 January 2000. Cohort censorship was defined by the first date of migration out of Wales/residency break, death, or the study endpoint on 31 December 2019 (figure 1). Within these constraints, the cohort is designed to be without selection bias and to achieve complete follow-up. WMC also provides a fully generalisable population sample against which findings from more selected samples may be compared.

**Figure 2:**
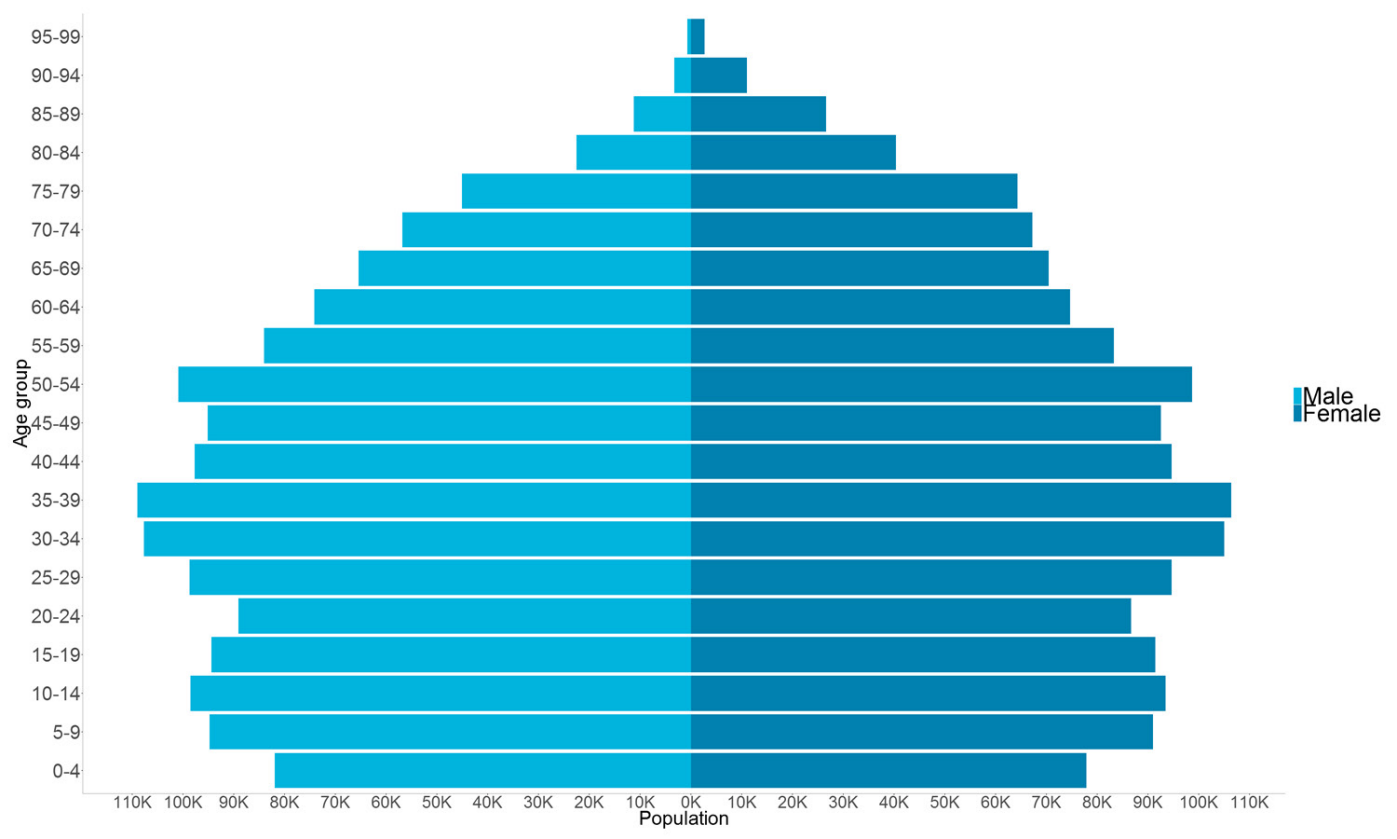
WMC pyramid for age (years) at cohort inception. WMC, Wales Multimorbidity e-Cohort.

**Figure 3:**
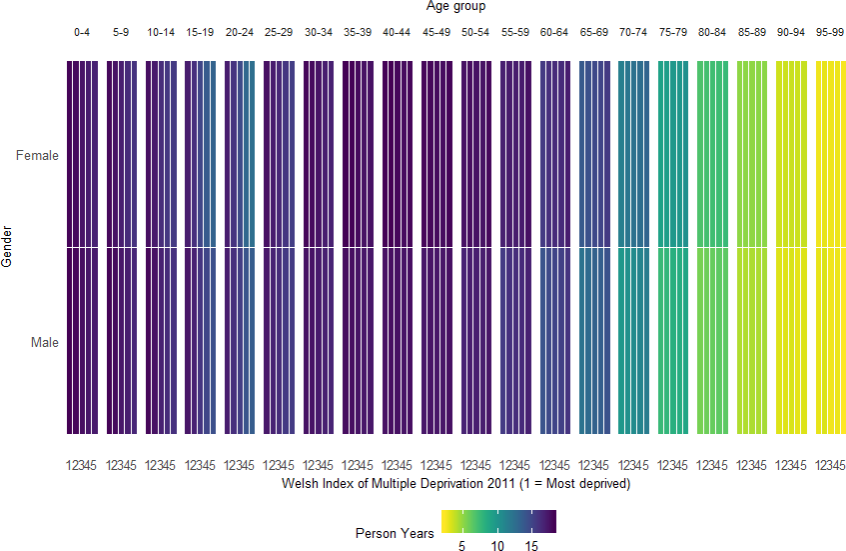
Heatmap of person years of WMC follow-up, by age group, sex and area-level deprivation at cohort inception. WMC, Wales Multimorbidity e-Cohort.

The WMC contains 2 902 101 individuals aged 0–99 at cohort start date with 46 million person years of follow-up available (table 1, figures 2 and 3, online supplemental appendix table A1 and A2). Individuals have a minimum of 1-day follow-up (cohort end date = 2 January 2000) and maximum of 20 years of follow-up (cohort end date = 31 December 2019).

10.1136/bmjopen-2020-047101.supp1 Supplementary data

**Table 1:** WMC baseline demographics

WMC characteristics	n (%)
Cohort size	2 902 101 (100)
Full coverage (1 January 2000–31 December 2019)	1 714 484 (59.08)
Residency break/emigration	643 472 (22.17)
Mortality	544 145 (18.75)
Primary care data available	2 470 874 (85.14)
Care home residency at cohort end	97 006 (3.34)
Mean age in years (range) at cohort start	39 (0–99)
Sex	
Female	1 472 113 (50.70)
Male	1 429 988 (49.30)
WIMD 2011 Quintile at cohort start	
1. Most deprived	605 203 (20.85)
2	589 479 (20.31)
3	584 039 (20.12)
4	557 319 (19.20)
5. Least deprived	566 061 (19.51)

WIMD, Welsh Index of Multiple Deprivation; WMC, Wales Multimorbidity e-Cohort.

The Heatmap in figure 3 visualises the person years of follow-up by age, sex and area level deprivation. The more years of follow-up available the darker the colour. Age is calculated at the cohort start, therefore, younger individuals will have more years of available follow-up compared with older individuals. On average, there are less person years of follow-up available for the least deprived 15–24 years old compared with their respective age group in other areas of Wales.

### Data sources

The WMC has used and combined anonymised health, social and environmental data held within the SAIL Databank (www.saildatabank.com).

The baseline characteristics for the WMC have been created using the Welsh Demographic Service Dataset (WDSD) and the Annual District Death Extract (ADDE) mortality registry data from the Office for National Statistics. The WDSD contains administrative information concerning the resident population of Wales that are registered to a Welsh General Practice, a free to use National Health Service (NHS) system at the point of primary care registration in the UK. The ADDE data contains information about the dates and causes of all deaths relating to residents in Wales, including those that died outside of Wales. SAIL holds general practitioner (GP) data for approximately 80% of the population with coverage extending to all local authorities in Wales. The Welsh Longitudinal General Practice data will be used to identify the subpopulation of individuals who are registered to a practice providing data to SAIL to identify which individuals have GP data present and avoid underestimation of conditions or severity of conditions not managed through hospital admission.

The Welsh Health Survey Dataset and the National Survey for Wales Dataset with data on well-being measures, social class, education, housing and wealth are available for 9905 and 33 295 cohort participants respectively.^24^

### Anonymised linkage fields

Linkage fields are used to anonymously link between data sources in the SAIL Databank and have been previously described elsewhere.^13 14 25^ SAIL uses a multiple encryption system in which a trusted third party, the NHS Wales Informatics Service, uniquely matches identities (NHS number, name, date of birth and residential address/ Unique Property Reference Number (UPRN)) and replaces these with unique identifiers. For individuals this is called an Anonymised Linkage Field (ALF) and Residential Anonymised Linkage Field (RALF) for pseudonymised residences before uploading data to SAIL.

### Demographic data

The cohort includes the following variables: ALF, age in years, sex, date of death, date of movement out of Wales, RALF at both cohort inception and cohort end and Care Home Anonymised Linkage Fields (CHALFs) at cohort end date. The CHALF was derived from a data extract from Care Inspectorate Wales in 2020 for all adult care home settings.^18^ Geographical variables associated with the RALF and CHALF include Lower layer Super Output Area (LSOA) 2001 at cohort inception and LSOA 2011 at cohort end. These have been mapped to the Welsh Index of Multiple Deprivation version 2011 and 2019, respectively, to derive socioeconomic deprivation quintiles and urban/rurality categories.^26 27^

### Health data

All admissions to hospital (inclusive of critical care admissions), outpatient, emergency department attendances treated in NHS hospitals as well as disease registries and laboratory test results data are available for cohort participants, GP data for diagnoses and treatments from SAIL providing practices are data for approximately 80% of the population.^28^

**Figure 4:**
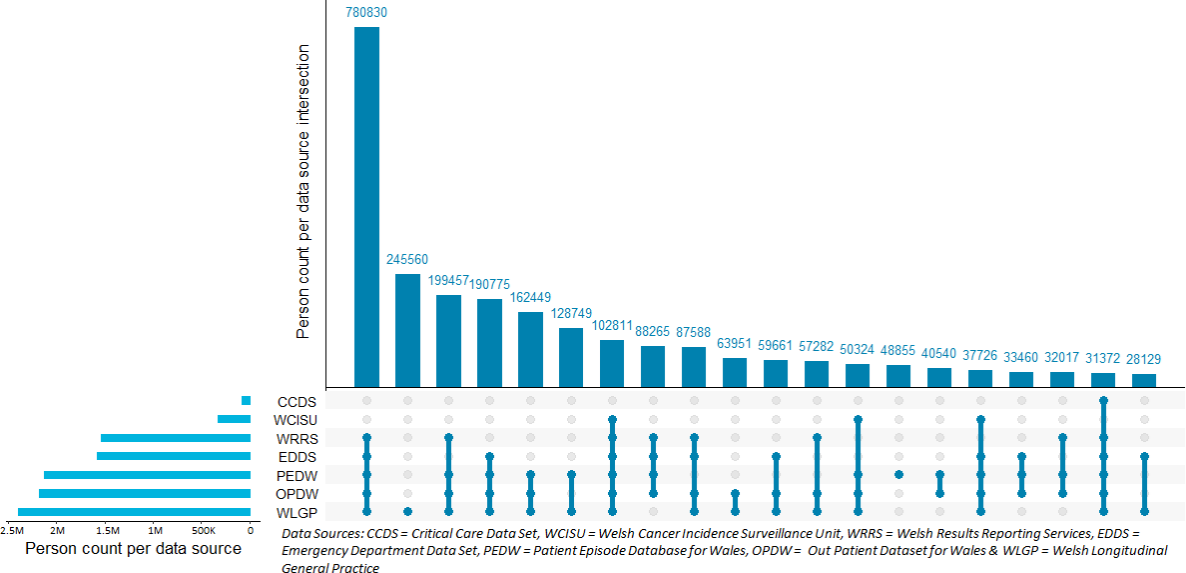
Number of WMC individuals utilising healthcare services recorded in multisource data sources, 20 most common combinations presented. WMC, Wales Multimorbidity e-Cohort.

All relevant health events recorded in clinical data sources will be joined onto the WMC to identify diagnosis of conditions, treatments and various significant heath events that occur across multisourced linked heath data per person (table 2 and figure 4).

**Table 2:** Clinical data sources available for the WMC

Data source	Period covered	No and percentage of WMC individuals with data
Critical Care Data Set	01-Jan-2007–31-Dec-2019	79 521 (2.7)
Welsh Cancer Incidence Surveillance Unit	01-Jan-2000–31-Dec-2016	328 792 (11.3)
Welsh Results Reporting Services	01-Jan-2015–10-Dec-2018	1 540 754 (53.1)
Emergency Department Data Set	01-Apr-2009–31-Dec-2019	1 579 665 (54.4)
Patient Episode Database for Wales)	01-Jan-2000–31-Dec-2019	2 129 384 (73.4)
Out Patient Dataset for Wales	01-Apr-2004–31-Dec-2019	2 177 081 (75.0)
Welsh Longitudinal General Practice	01-Jan-2000–31-Dec-2019	2 400 313 (82.7)

Please note clinical data sources will be updated on a monthly/quarterly basis.

WMC, Wales Multimorbidity e-Cohort.

The Upset plot in figure 4 demonstrates the number of WMC participants that have interacted with the various healthcare settings from 1 January 2000 to their cohort censorship end date.^29^ For example, 780 830 (26.9%) individuals have used GP, inpatient, outpatient and emergency department services as well as had at least one laboratory test within their WMC coverage.

### Phenotyping the e-cohort

Published comorbidity indices and phenotype code lists (International Classification of Diseases 10th revision (ICD-10), OPCS Classification of Interventions and Procedures version 4 (OPCS4) and primary care Read Codes version 2) will be used to measure and conceptualise multimorbidity. These include those created by: CALIBER initiative; Charlson Comorbidity Index; Common Mental Disorders (CMD); Elixhauser Comorbidity Index; GBD Study and the NHS Quality and Outcomes Framework (QOF).^30–41^ Diagnostic codes relating to HIV will not be included in any outputs to conform with SAIL policies. They are part of the list of redacted codes not allowed to be used for research using the data.^42^ All ICD-10 and OPCS4 codes provided at the three character level were expanded to include all children terms.

#### CALIBER

Phenotyping algorithms created from the CALIBER resource using ICD-10, OPCS4 and Read Codes will be used to identify 300 physical and mental health conditions recorded in both primary and secondary healthcare.^31 39^

There are 1645 distinct ICD-10 codes (at three and four-character level) for 300 conditions, however, when capturing all ICD-10 codes to include variation in coding entry (eg, C796– instead of C796) and expanding the code list to the four-character level (F200 instead of F20), there are 3702 distinct ICD-10 codes (at the four-character level) recorded in the inpatient data. This is important to note as to link solely on standardised codes would result in loss of information and potential reporting of false negatives.

There are 587 distinct OPCS4 codes (at three and four-character level) for 28 conditions and 8588 distinct Read Codes (at the five-character level) for 275 conditions.

#### Charlson Comorbidity Index

The Aylin and Bottle Charlson amended ICD-10 code list will be used for inpatient diagnosis and the Metcalfe *et al*^33^ Charlson Read Code list will be utilised for primary care recorded diagnosis.^32 33^

The ICD-10 codes have been taken from the pool of diagnosis codes recorded within hospital admissions data, containing 1024 distinct codes (at the four-character level) for 16 conditions. The GP data contains 4545 distinct Read Codes at the five-character level.

#### Common mental disorders

The John *et al* validated algorithm will be used to identify CMD in GP data.^30 40 41^ The algorithm has used a combination of diagnosis, treatment and symptoms Read Codes in identifying CMD. Individuals with CMD are identified as either having a historical diagnosis code, currently treated or, having a current diagnosis/current symptom code. There are 89 distinct diagnosis codes, 15 symptom codes and 601 treatment codes.

#### Elixhauser Comorbidity Index

The Quan et al (2005) Elixhauser ICD-10 code list will be utilised for inpatient diagnosis and the Metcalfe *et al*^33^ Elixhauser Read Code list will be utilised for primary care recorded diagnosis.^33 34^

The ICD-10 codes have been taken from the pool of diagnosis codes recorded within hospital admissions data and contains 1423 distinct codes (at the four-character level) for 30 conditions. The general practice data contains 6074 distinct Read codes at the five-character level.

#### GBD Study

The GBD 2019 ICD-10 codes will be used to identify 130 health conditions in secondary healthcare data. There are 3497 distinct ICD-10 codes at the three and four-character level.^38^

#### Quality Outcome Framework

The QOF conditions business rule V.38 will be used to identify 18 health conditions in primary care data.^35^ The 18 conditions are asthma, atrial fibrillation, obesity, coronary heart disease, chronic obstructive pulmonary disease, cancer, chronic kidney disease, dementia, depression, diabetes, epilepsy, heart failure, hypertension, learning difficulties, peripheral arterial disease, rheumatoid arthritis, serious mental illness and stroke. There are 2275 distinct Read Codes available at the five-character level for the 18 QOF conditions.

### Statistical analysis

The WMC provides an accessible research ready data asset to further understanding of multimorbidity through the use of biostatistical and machine learning approaches. Our collaborative team will work across a number of projects to develop and evaluate statistical and machine learning algorithms to address the following broad analytical challenges:

- What is the prevalence of multimorbidity in the WMC, and how does prevalence of multimorbidity change over time?
- What are common clusters of multimorbidity in the WMC, and how do they correspond to or differ from, common clusters of multimorbidity identified in other datasets?
- Which clusters of multimorbidity occur less frequently than one would expect based on the prevalence of their constituent conditions?
- How does multimorbidity develop across the life course (ie, trajectories)?
- What are the biological, psychological and social determinants of different clusters and trajectories of multimorbidity?
- Which clusters and trajectories of multimorbidity are associated with poor health outcomes?
- Which clusters and trajectories of multimorbidity are associated with high service utilisation?
- Does multimorbidity in specific groups (eg, patients with musculoskeletal conditions) differ from multimorbidity in general?

The overarching aim is to evaluate and provide new multimorbidity phenotypes and algorithms to the NHS and research communities to support prevention, healthcare planning and the management of individuals with multimorbidity.

We will draw on both methods from statistics (eg, regression analysis, longitudinal mixed models, multiple correspondence analysis, factor analysis,^43^ multistate models and latent class analysis) and machine learning (eg, k-means clustering, semantic similarity clustering, market basket analysis, network models^44^ and deep learning). We will use resampling methods to assess the stability of identified multimorbidity clusters and develop visualisation techniques to summarise multimorbidity clusters and their associations with risk factors and outcomes.

Analyses will be coded in R, WinBUGS, and Python and made available to WMC users via a Git library to maximise transparency and reproducibility.^45^

### Patient and public involvement

The proposal to develop WMC was submitted to the independent Information Governance Review Panel (IGRP) that includes members of the public (IGRP Project: 0911). We worked with this group to refine the study protocol. The scientific steering group includes two members of the public who have contributed to this paper. The HNIMR has a work package on patient and public involvement with a panel drawn from across the UK which meets to discuss the research work and feed into the research and dissemination plans.

## Ethics and dissemination

The use of deidentified data in SAIL complies with National Research Ethics Service (NRES) guidance.^46^ Applications to use data held within the SAIL Databank, an ISO: 27001 and UK Statistics Authority (UKSA) Digital Economy Act (DEA) accredited Trusted Research Environment, must first be approved by the independent IGRP. This panel contains individuals with expertise in data governance and protection, including the Chair of the Wales NRES Committee, Caldicott Guardians and members of the public. WMC was approved by IGRP on 26 June 2019.

Findings from this study will be disseminated widely through a variety of routes, including to health policy and NHS leads across UK, the AMS and the Royal Colleges, as well as traditional scientific outlets. The team includes NHS clinicians and informaticians to allow for early NHS adoption of useful findings. Members of the public embedded in the team will create plain English summaries and lead at public facing meetings.

## Supplementary Material

Reviewer comments

Author's manuscript
