# Supplementary material for: Protocol for the development of the Wales Multimorbidity e-Cohort (WMC): data sources and methods to construct a population-based research platform to investigate multimorbidity
Source: BMJ Open. 2021 Jan 19;11(1):e047101. doi: 10.1136/bmjopen-2020-047101 (PMC7817800; doi:10.1136/bmjopen-2020-047101)
Supplement: Supplementary data [file bmjopen-2020-047101supp001.pdf]

Appendix

Table A1: WMC participants categorised by age group and sex at cohort start

Age group	Sex	Count	Percentage
00-04	Male	81,915	2.82
00-04	Female	77,873	2.68
05-09	Male	94,737	3.26
05-09	Female	90,940	3.13
10-14	Male	98,466	3.39
10-14	Female	93,447	3.22
15-19	Male	94,345	3.25
15-19	Female	91,440	3.15
20-24	Male	89,037	3.07
20-24	Female	86,666	2.99
25-29	Male	98,622	3.40
25-29	Female	94,592	3.26
30-34	Male	107,671	3.71
30-34	Female	104,986	3.62
35-39	Male	108,964	3.75
35-39	Female	106,312	3.66
40-44	Male	97,637	3.36
40-44	Female	94,599	3.26
45-49	Male	95,071	3.28
45-49	Female	92,478	3.19
50-54	Male	100,866	3.48
50-54	Female	98,606	3.40
55-59	Male	83,949	2.89
55-59	Female	83,210	2.87
60-64	Male	74,115	2.55
60-64	Female	74,591	2.57
65-69	Male	65,354	2.25
65-69	Female	70,389	2.43
70-74	Male	56,746	1.96
70-74	Female	67,227	2.32
75-79	Male	45,027	1.55
75-79	Female	64,274	2.21
80-84	Male	22,441	0.77
80-84	Female	40,344	1.39
85-89	Male	11,184	0.39
85-89	Female	26,540	0.91
90-94	Male	3,208	0.11
90-94	Female	10,951	0.38
95-99	Male	633	0.02
95-99	Female	2,648	0.09

Table A2: WMC average person years of follow up, categorised by age group, sex and WIMD 2011 at cohort start

Age group	Sex	WIMD 2011 quintiles	Average Pys
00-04	Male	1	18.16
00-04	Male	2	17.93
00-04	Male	3	17.63
00-04	Male	4	17.28
00-04	Male	5	17.11
05-09	Male	1	18.06
05-09	Male	2	17.81
05-09	Male	3	17.26
05-09	Male	4	16.91
05-09	Male	5	16.50
10-14	Male	1	17.93
10-14	Male	2	17.46
10-14	Male	3	16.76
10-14	Male	4	16.24
10-14	Male	5	15.98
15-19	Male	1	17.30
15-19	Male	2	16.73
15-19	Male	3	15.75
15-19	Male	4	14.89
15-19	Male	5	14.34
20-24	Male	1	16.96
20-24	Male	2	16.04
20-24	Male	3	15.29
20-24	Male	4	14.03
20-24	Male	5	13.59
25-29	Male	1	17.18
25-29	Male	2	16.71
25-29	Male	3	16.22
25-29	Male	4	15.57
25-29	Male	5	15.47
30-34	Male	1	17.44
30-34	Male	2	17.41
30-34	Male	3	17.11
30-34	Male	4	16.82
30-34	Male	5	16.60
35-39	Male	1	17.66
35-39	Male	2	17.63
35-39	Male	3	17.41
35-39	Male	4	17.27

35-39	Male	5	17.22
40-44	Male	1	17.50
40-44	Male	2	17.63
40-44	Male	3	17.55
40-44	Male	4	17.40
40-44	Male	5	17.57
45-49	Male	1	17.23
45-49	Male	2	17.39
45-49	Male	3	17.28
45-49	Male	4	17.24
45-49	Male	5	17.48
50-54	Male	1	16.68
50-54	Male	2	16.96
50-54	Male	3	16.89
50-54	Male	4	16.91
50-54	Male	5	17.30
55-59	Male	1	15.46
55-59	Male	2	16.03
55-59	Male	3	16.20
55-59	Male	4	16.31
55-59	Male	5	16.78
60-64	Male	1	14.07
60-64	Male	2	14.64
60-64	Male	3	14.96
60-64	Male	4	15.19
60-64	Male	5	15.80
65-69	Male	1	12.14
65-69	Male	2	12.70
65-69	Male	3	13.24
65-69	Male	4	13.46
65-69	Male	5	14.21
70-74	Male	1	9.73
70-74	Male	2	10.23
70-74	Male	3	10.59
70-74	Male	4	11.02
70-74	Male	5	11.58
75-79	Male	1	7.46
75-79	Male	2	7.73
75-79	Male	3	8.14
75-79	Male	4	8.29
75-79	Male	5	8.79
80-84	Male	1	5.55
80-84	Male	2	5.82
80-84	Male	3	6.02

80-84	Male	4	6.24
80-84	Male	5	6.30
85-89	Male	1	4.20
85-89	Male	2	4.18
85-89	Male	3	4.28
85-89	Male	4	4.27
85-89	Male	5	4.41
90-94	Male	1	3.09
90-94	Male	2	3.07
90-94	Male	3	3.22
90-94	Male	4	2.95
90-94	Male	5	3.13
95-99	Male	1	2.87
95-99	Male	2	3.19
95-99	Male	3	2.64
95-99	Male	4	2.77
95-99	Male	5	2.32
00-04	Female	1	18.03
00-04	Female	2	17.82
00-04	Female	3	17.37
00-04	Female	4	16.99
00-04	Female	5	16.73
05-09	Female	1	17.84
05-09	Female	2	17.44
05-09	Female	3	16.89
05-09	Female	4	16.27
05-09	Female	5	15.92
10-14	Female	1	17.57
10-14	Female	2	17.09
10-14	Female	3	16.20
10-14	Female	4	15.60
10-14	Female	5	15.51
15-19	Female	1	16.93
15-19	Female	2	16.08
15-19	Female	3	14.88
15-19	Female	4	13.68
15-19	Female	5	13.21
20-24	Female	1	16.94
20-24	Female	2	15.81
20-24	Female	3	14.61
20-24	Female	4	12.89
20-24	Female	5	12.48
25-29	Female	1	17.53
25-29	Female	2	16.99

25-29	Female	3	16.47
25-29	Female	4	15.77
25-29	Female	5	15.34
30-34	Female	1	17.94
30-34	Female	2	17.77
30-34	Female	3	17.39
30-34	Female	4	16.94
30-34	Female	5	16.77
35-39	Female	1	18.18
35-39	Female	2	18.12
35-39	Female	3	17.78
35-39	Female	4	17.47
35-39	Female	5	17.49
40-44	Female	1	18.11
40-44	Female	2	18.16
40-44	Female	3	17.91
40-44	Female	4	17.71
40-44	Female	5	17.92
45-49	Female	1	17.92
45-49	Female	2	17.93
45-49	Female	3	17.82
45-49	Female	4	17.66
45-49	Female	5	17.97
50-54	Female	1	17.49
50-54	Female	2	17.69
50-54	Female	3	17.49
50-54	Female	4	17.44
50-54	Female	5	17.87
55-59	Female	1	16.79
55-59	Female	2	17.09
55-59	Female	3	17.00
55-59	Female	4	17.06
55-59	Female	5	17.54
60-64	Female	1	15.53
60-64	Female	2	16.04
60-64	Female	3	16.23
60-64	Female	4	16.28
60-64	Female	5	16.96
65-69	Female	1	13.76
65-69	Female	2	14.28
65-69	Female	3	14.70
65-69	Female	4	14.92
65-69	Female	5	15.52
70-74	Female	1	11.43

70-74	Female	2	11.93
70-74	Female	3	12.27
70-74	Female	4	12.57
70-74	Female	5	13.12
75-79	Female	1	9.06
75-79	Female	2	9.35
75-79	Female	3	9.70
75-79	Female	4	9.82
75-79	Female	5	10.25
80-84	Female	1	6.78
80-84	Female	2	7.07
80-84	Female	3	7.19
80-84	Female	4	7.26
80-84	Female	5	7.50
85-89	Female	1	4.98
85-89	Female	2	5.02
85-89	Female	3	4.97
85-89	Female	4	5.07
85-89	Female	5	5.07
90-94	Female	1	3.45
90-94	Female	2	3.53
90-94	Female	3	3.61
90-94	Female	4	3.55
90-94	Female	5	3.66
95-99	Female	1	2.66
95-99	Female	2	2.87
95-99	Female	3	2.70
95-99	Female	4	2.75
95-99	Female	5	2.48
