# Supplementary material for: Protocol for the development of the Wales Multimorbidity e-Cohort (WMC): data sources and methods to construct a population-based research platform to investigate multimorbidity
Source: BMJ Open. 2021 Jan 19;11(1):e047101. doi: 10.1136/bmjopen-2020-047101 (PMC7817800; doi:10.1136/bmjopen-2020-047101)
Supplement: Author's manuscript [file bmjopen-2020-047101.draft_revisions.pdf]

BMJ Open is committed to open peer review. As part of this commitment we make the peer review history of every article we publish publicly available.

When an article is published we post the peer reviewers' comments and the authors' responses online. We also post the versions of the paper that were used during peer review. These are the versions that the peer review comments apply to.

The versions of the paper that follow are the versions that were submitted during the peer review process. They are not the versions of record or the final published versions. They should not be cited or distributed as the published version of this manuscript.

BMJ Open is an open access journal and the full, final, typeset and author-corrected version of record of the manuscript is available on our site with no access controls, subscription charges or pay-per-view fees (http://bmjopen.bmj.com).

If you have any questions on BMJ Open's open peer review process please email info.bmjopen@bmj.com

BMJ Open

The Wales Multi-morbidity e-Cohort (WMC): data sources and methods to construct a population-based research platform to investigate multi-morbidity.

Journal:	*BMJ Open*
Manuscript ID	bmjopen-2020-047101
Article Type:	Protocol
Date Submitted by the Author:	18-Nov-2020
Complete List of Authors:	Lyons, Jane; Swansea University Medical School,<br>Akbari, Ashley; Swansea University Medical School,<br>Agrawal, Utkarsh; University of St Andrews, School of Medicine<br>Harper, Gill; Queen Mary University of London<br>Azcoaga-Lorenzo, Amaya; University of Saint Andrews School of Medicine, Division of Population and Behavioural Sciences<br>Bailey, Rowena; Swansea University Medical School, Population Data Science<br>Rafferty, James; Swansea University Medical School<br>Watkins, Alan; Swansea University, College of Medicine<br>Fry, Richard; Swansea University, Medical School<br>McCowan, Colin ; University of St Andrews<br>Dezateux, Carol; Queen Mary University of London, Centre for Primary Care and Public Health<br>Robson, John; Barts and The London School of Medicine and Dentistry, Queen Mary University of London, Centre for Primary Care and Public Health<br>Peek, N; Health e-Research Centre, Institute of Population Health, University of Manchester<br>Holmes, Chris; Oxford University<br>Denaxas, S; University College London<br>Owen, R; University of Leicester<br>Abrams, Keith; University of Leicester, Biostatistics Research Group, Department of Health Sciences<br>John, Ann; Swansea University<br>OReilly, Dermot; Queens University Belfast, Epidemiology and Public Health<br>Richardson , Sylvia; MRC Biostatistics Unit, Department of Epidemiology and Public Health<br>Hall, Marlous ; Leeds<br>Gale, Chris; University of Leeds<br>Davies, Jan; Swansea University,<br>Davies, Chris; Swansea University,<br>Cross, Lynsey; Swansea University Medical School, Population Data Science<br>Gallacher, John; Oxford University<br>Chess, James; Swansea Bay University Health Board, Renal Unit<br>Brookes, Anthony; University of Leicester<br>Lyons, Ronan; Swansea University, Swansea Clinical School

Keywords:	PUBLIC HEALTH, EPIDEMIOLOGY, Health policy < HEALTH SERVICES ADMINISTRATION & MANAGEMENT, PRIMARY CARE, GERIATRIC MEDICINE

SCHOLARONE™
Manuscripts

BMJ

I, the Submitting Author has the right to grant and does grant on behalf of all authors of the Work (as defined in the below author licence), an exclusive licence and/or a non-exclusive licence for contributions from authors who are: i) UK Crown employees; ii) where BMJ has agreed a CC-BY licence shall apply, and/or iii) in accordance with the terms applicable for US Federal Government officers or employees acting as part of their official duties; on a worldwide, perpetual, irrevocable, royalty-free basis to BMJ Publishing Group Ltd ("BMJ") its licensees and where the relevant Journal is co-owned by BMJ to the co-owners of the Journal, to publish the Work in this journal and any other BMJ products and to exploit all rights, as set out in our licence.

The Submitting Author accepts and understands that any supply made under these terms is made by BMJ to the Submitting Author unless you are acting as an employee on behalf of your employer or a postgraduate student of an affiliated institution which is paying any applicable article publishing charge ("APC") for Open Access articles. Where the Submitting Author wishes to make the Work available on an Open Access basis (and intends to pay the relevant APC), the terms of reuse of such Open Access shall be governed by a Creative Commons licence – details of these licences and which Creative Commons licence will apply to this Work are set out in our licence referred to above.

Other than as permitted in any relevant BMJ Author's Self Archiving Policies, I confirm this Work has not been accepted for publication elsewhere, is not being considered for publication elsewhere and does not duplicate material already published. I confirm all authors consent to publication of this Work and authorise the granting of this licence.

For peer review only

The Wales Multi-morbidity e-Cohort (WMC): data sources and methods to construct a population-based research platform to investigate multi-morbidity.

Jane Lyons, Data Science Building, Population Data Science, Swansea University Medical School, Singleton Park, Swansea, SA2 8PP, UK, J.Lyons@Swansea.ac.uk, 01792 513028 (corresponding author)

Ashley Akbari, Swansea University, Swansea, UK

Dr Utkarsh Agrawal, University of St Andrews, St Andrews, UK

Dr Gill Harper, Queen Mary University of London, London, UK

Dr Amaya Azcoaga-Lorenzo, University of St Andrews, St Andrews, UK

Rowena Bailey, Swansea University, Swansea, UK

Dr James Rafferty, Swansea University, Swansea, UK

Professor Alan Watkins, Swansea University, Swansea, UK

Dr Richard Fry, Swansea University, Swansea, UK

Professor Colin McCowan, University of St Andrews, St Andrews, UK

Professor Carol Dezateux, Queen Mary University of London, London, UK

Dr John P Robson, Queen Mary University of London, London, UK

Professor Niels Peek, University of Manchester, Manchester, UK

Professor Chris Holmes, University of Oxford, Oxford, UK

Professor Spiros Denaxas, University College London, London, UK

Dr Rhiannon Owen, University of Leicester, Leicester, UK

Professor Keith R Abrams, University of Leicester, Leicester, UK

Professor Ann John, Swansea University, Swansea, UK

Professor Dermot O'Reilly, Queens University, Belfast, UK

Professor Sylvia Richardson, Cambridge University, Cambridge, UK

Dr Marlous Hall, University of Leeds, Leeds, UK

Professor Chris P Gale, University of Leeds, Leeds UK

Jan Davies, Member of public, Swansea, UK

Chris Davies, Member of public, Swansea, UK

Lynsey Cross, Swansea University, Swansea, UK

Professor John Gallacher, Oxford University, Oxford, UK

Dr James Chess, Swansea Bay University Health Board, Swansea, UK

Professor Anthony J Brookes, University of Leicester, Leicester, UK

Professor Ronan A Lyons, Swansea University, Swansea, UK

Keywords: Multi-morbidity, data linkage, epidemiology, public health, evaluation

For peer review only

Word count: 2842/4000

ABSTRACT

Introduction

Multi-morbidity is widely recognised as the presence of two or more concurrent long-term conditions, but remains a poorly understood global issue despite increasing in prevalence.

We have created the Wales Multi-morbidity e-Cohort (WMC) to provide an accessible research ready data asset to further the understanding of multi-morbidity. Our objectives are to create a platform to support research which would help to understand prevalence, trajectories and determinants in multi-morbidity, characterise clusters that lead to highest burden on individuals and healthcare services, and evaluate and provide new multi-morbidity phenotypes and algorithms to the NHS and research communities to support prevention, healthcare planning and the management of individuals with multi-morbidity.

Methods and analysis

The WMC has been created and derived from multi-sourced demographic, administrative and electronic health record (EHR) data relating to the Welsh population in the Secure Anonymised Information Linkage (SAIL) Databank. The WMC consists of 2.9 million people alive and living in Wales on the 1st January 2000 with follow up until 31st December 2019, Welsh residency break or death. Published comorbidity indices and phenotype code lists will be used to measure and conceptualise multi-morbidity.

Study outcomes will include: a) a description of multi-morbidity using published data phenotype algorithms/ontologies, b) investigation of the associations between baseline demographic factors and multi-morbidity c) identification of temporal trajectories of clusters of conditions and multi-morbidity, d) investigation of multi-morbidity clusters with poor outcomes such as mortality and high healthcare service utilisation.

Ethics and dissemination

The SAIL Databank independent Information Governance Review Panel (IGRP) has approved this study (SAIL Project: 0911). Study findings will be presented to policy groups, public meetings, national and international conferences, and published in peer-reviewed journals.

For peer review only

Strengths and limitations of this study

- Creation and access to a multi-sourced population based, deeply phenotyped e-cohort.
- Future use of this resource removes need for data management and cleaning of source data, accelerating research and which could also support efforts for reproducibility of results.
- Variety of individual and household level data on demography, health status, health care utilisation, both primary and secondary healthcare, and mortality to support a wide range of analytical approaches to addressing scientific questions.
- Input from multiple disciplines and institutions from across all four nations of the United Kingdom to help understand, measure and address multi-morbidity.
- Routine data does not capture data on some important aspects, such as quality of life.

For peer review only

INTRODUCTION

Multi-morbidity is defined by the UK's Academy of Medical Sciences (AMS) and the World Health Organisation as the presence of two or more concurrent long-term conditions, which is a global and growing phenomenon.[1,2] Multi-morbidity is more prevalent in older individuals and associated with high healthcare utilisation and mortality, but with large numbers of patients of all age suffering from multi-morbidity.[3-6] With an aging population, it is estimated that two in three people in England aged 65 years or over will experience multi-morbidity by 2035 and nearly one fifth will have complex multi-morbidity (4 or more conditions).[7]

Much of what is known about multi-morbidity is based on a limited and fragmented knowledge base, largely derived from studies of older people in high-income countries or hospital populations.[1,8] The 2018 AMS report concluded that multi-morbidity is an unhelpful term implying random assortment of disease when it often refers to clusters of specific diseases. Once identified, these disease clusters can be addressed specifically through research, healthcare policy development and service delivery.[1,9] The identification of previously unrecognised disease clusters may also provide biological and clinical insights into their aetiology, prevention and treatment. The AMS report identified specific research gaps and proposed a list of priorities (Figure 1). Several can be addressed through a combination of health data science, epidemiology and statistics, and by exploiting the potential from creating deeply phenotyped cohorts from population and clinical data sources.

Figure 1: The Academy of Medical Sciences identified research gaps.

- The scale and nature of multi-morbidity and how it is changing over time.
- Which clusters of conditions cause the biggest problems for patients.
- The causes of the most common clusters including links with sex, ethnicity, income and lifestyle.
- The best ways to prevent the patients developing multi-morbidity, and whether this requires different approaches to just preventing individual conditions.
- How doctors can increase the benefits and reduce the risks of treatment for patients with multi-morbidity.
- How to organise healthcare systems to deal with multi-morbidity more effectively and how best to use digital technology in caring for patients.

Responding to this agenda, we created a privacy protecting total population electronic cohort - the Wales Multi-morbidity e-Cohort (WMC) - as a platform to study these issues in depth, collaborating with scientists

For peer review only - http://bmjopen.bmj.com/site/about/guidelines.xhtml

from many different institutions and disciplines, clinicians, and members of the public from across the UK to create a broader team science approach.

The objectives of this work are to understand prevalence, trajectories and determinants of multi-morbidity, and identify clusters causing the greatest health care burden. The WMC will also contribute data on incidence, prevalence and burden to the Global Burden of Diseases Study,[10,11] and provide new multi-morbidity phenotypes to e-cohorts with local participants, and phenotyping algorithms to many e-cohorts that utilise routine data.[12]

We expect that findings from these analyses will provide evidence to health policy leads in order to support prevention and the complex healthcare planning and management of multi-morbid individuals. Members of the public are embedded in the research team to ensure the resource focuses on issues of concern to the public.

This paper describes the creation of the WMC and the statistical approaches that will be developed to support the diverse research objectives.

METHODS

The WMC was developed by linking multiple routinely collected population and clinical data sources on the population of Wales from 2000-2019. We used the privacy-protecting Secure Anonymised Information Linkage (SAIL) Databank, to contribute to the Health Data Research UK National Implementation Multi-morbidity Resource (HNIMR) project, and extended to 2020 for the MRC funded Welsh Multi-morbidity Machine Learning (WMML) project .[13,14] SAIL is one of the most comprehensive, privacy protecting, linked data Trusted Research Environments (TRE) in the UK. SAIL utilising data from many different sources and providing linkage at individual and household level.[15] It has supported many different study designs, including, large-scale community-based or clinical condition-based observational studies, disease surveillance, evaluation of natural experiments of environmental interventions, embedded trials, and the Dementias Platform UK.[16-23]

Cohort design and characteristics

WMC is a clearly defined complete population cohort. Cohort entry includes all residents in Wales, alive and living on the 1st January 2000. Cohort censorship was defined by the first date of migration out of Wales/residency break, death or the study endpoint on 31st December 2019 (Figure 2). Within these constraints, the cohort is designed to be without selection bias and to achieve complete follow-up. WMC also provides a fully generalisable population sample against which findings from more selected samples may be compared.

For peer review only

The WMC contains 2,902,101 individuals aged 0-99 at cohort start date with 46 million person years of follow up available (Table 1, Figures 3 & 4, Appendix Table A1 & A2). Individuals have a minimum of 1-day follow up (cohort end date = 2nd January 2000) and maximum of 20-years of follow up (cohort end date = 31st December 2019).

Table 1: WMC baseline demographics

WMC characteristics	n	%
Cohort size	2,902,101	100
Full coverage (01-01-2000 – 31-12-2019)	1,714,484	59.08
Residency break/Emigration	643,472	22.17
Mortality	544,145	18.75
Primary care data available	2,470,874	85.14
Care home residency at cohort end	97,006	3.34
Mean age in years (range) at cohort start	39 (0-99)	
Sex		
Female	1,472,113	50.60
Male	1,436,988	49.40
WIMD 2011 Quintile at cohort start		
1. Most Deprived	605,203	20.85
2	589,479	20.31
3	584,039	20.12
4	557,319	19.20
5. Least Deprived	566,061	19.51

The Heatmap in Figure 4 visualises the person years of follow up by age, sex and area level deprivation. The more years of follow up available the darker the colour. Age is calculated at the cohort start, therefore, younger individuals will have more years of available follow up compared to older individuals. On average, there are less person years of follow up available for the least deprived 15-24 year olds compared to their respective age group in other areas of Wales.

Data Sources

The WMC has utilised and combined anonymised health, social and environmental data held within the SAIL Databank (www.saildatabank.com).

For peer review only

The baseline characteristics for the WMC have been created using the Welsh Demographic Service Dataset (WDSD) and the Annual District Death Extract (ADDE) mortality registry data from the Office for National Statistics. The WDSD contains administrative information concerning the resident population of Wales that are registered to a Welsh General Practice, a free to use National Health Service (NHS) system at the point of primary care registration in the UK. The ADDE data contains information about the dates and causes of all deaths relating to residents in Wales, including those that died outside of Wales. SAIL holds GP data for approximately 80% of the population with coverage extending to all local authorities in Wales. The Welsh Longitudinal General Practice (WLGP) data will be used to identify the sub-population of individuals who are registered to a practice providing data to SAIL to identify which individuals have GP data present and avoid underestimation of conditions or severity of conditions not managed through hospital admission.

The Welsh Health Survey Dataset (WHSD) and the National Survey for Wales Dataset (NSWD) with data on wellbeing measures, social class, education, housing and wealth are available for 9,905 and 33,295 cohort participants respectively. [24]

Anonymised Linkage Fields

Linkage fields are used to anonymously link between data sources in the SAIL Databank and have been previously described elsewhere.[13,14,25] SAIL utilises a multiple encryption system in which a trusted third party, the National Health Service (NHS) Wales Informatics Service (NWIS), uniquely matches identities (NHS number, name, date of birth, and residential address/UPRN) and replaces these with unique identifiers. For individuals this is called an Anonymised Linkage Field (ALF) and Residential Anonymised Linkage Field (RALF) for pseudonymised residences before uploading data to SAIL.

Demographic Data

The cohort includes the following variables: Anonymised Linkage Field (ALF), age in years, sex, date of death, date of movement out of Wales, RALF at both cohort inception and cohort end and Care Home Anonymised Linkage Fields (CHALFs) at cohort end date. The CHALF was derived from a data extract from Care Inspectorate Wales in 2020 for all adult care home settings.[18] Geographical variables associated with the RALF and CHALF include Lower layer Super Output Area (LSOA) 2001 at cohort inception and LSOA 2011 at cohort end. These have been mapped to the Welsh Index of Multiple Deprivation (WIMD) version 2011 and 2019 respectively to derive socioeconomic deprivation quintiles and urban/rurality categories.[26,27]

Health Data

All admissions to hospital (inclusive of critical care admissions), outpatient, Emergency Department attendances treated in NHS hospitals as well as disease registries and laboratory test results data are available for cohort participants, GP data for diagnoses and treatments from SAIL providing practices are data for approximately 80% of the population.[28]

For peer review only

All relevant health events recorded in clinical data sources will be joined onto the WMC to identify diagnosis of conditions, treatments and various significant heath events that occur across multi-sourced linked heath data per person (Table 2 &Figure 5).

Table 2: Clinical data sources available for the WMC.

Data source	Period covered	Number and percentage of WMC individuals with data
Critical Care Data Set (CCDS)	01-01-2007 – 31-12-2019	79,521 (2.7%)
Welsh Cancer Incidence Surveillance Unit (WCISU)	01-01-2000 – 31-12-2016	328,792 (11.3%)
Welsh Results Reporting Services (WRRS)	01-01-2015 – 10-12-2018	1,540,754 (53.1%)
Emergency Department Data Set (EDDS)	01-04-2009 – 31-12-2019	1,579,665 (54.4%)
Patient Episode Database for Wales (PEDW)	01-01-2000 – 31-12-2019	2,129,384 (73.4%)
Out Patient Dataset for Wales (OPDW)	01-04-2004 – 31-12-2019	2,177,081 (75.0%)
Welsh Longitudinal General Practice (WLGP)	01-01-2000 – 31-12-2019	2,400,313 (82.7%)
Please note clinical data sources will be updated on a monthly/quarterly basis		

The Upset plot in Figure 5 demonstrates the number of WMC participants that have interacted with the various health care settings from 1st January 2000 to their cohort censorship end date.[29] For example, 780,830 (26.9%) individuals have utilised GP, inpatient, outpatient and emergency department services as well as had at least one laboratory test within their WMC coverage.

Phenotyping the e-cohort

Published comorbidity indices and phenotype code lists (International Classification of Diseases 10th revision (ICD-10), OPCS Classification of Interventions and Procedures version 4 (OPCS4) and primary care Read Codes version 2) will be used to measure and conceptualise multi-morbidity. These include those created by: CALIBER initiative; Charlson Comorbidity Index; Common Mental Disorders (CMD); Elixhauser Comorbidity

For peer review only

Index; Global Burden of Disease Study; and the NHS Quality and Outcomes Framework (QOF).[30-41] Diagnostic codes relating to HIV will not be included in any outputs to conform with SAIL policies. They are part of the list of redacted codes not allowed to be used for research using the data.[42] All ICD-10 and OPCS4 codes provided at the three character level were expanded to include all children terms.

1. CALIBER

Phenotyping algorithms created from the CALIBER resource using ICD-10, OPCS4 and Read Codes will be utilised to identify 300 physical and mental health conditions recorded in both primary and secondary healthcare.[31,39]

There are 1,645 distinct ICD-10 codes (at three and four-character level) for 300 conditions, however, when capturing all ICD-10 codes to include variation in coding entry (e.g. C796– instead of C796) and expanding the code list to the four-character level (F200 instead of F20), there are 3,702 distinct ICD-10 codes (at the four-character level) recorded in the inpatient data. This is important to note as to link solely on standardised codes would result in loss of information and potential reporting of false negatives.

There are 587 distinct OPCS4 codes (at three and four-character level) for 28 conditions and 8,588 distinct Read Codes (at the five-character level) for 275 conditions.

2. Charlson Comorbidity Index

The Aylin and Bottle Charlson amended ICD-10 code list will be utilised for inpatient diagnosis and the Metcalfe et al (2019) Charlson Read Code list will be utilised for primary care recorded diagnosis.[32,33]

The ICD-10 codes have been taken from the pool of diagnosis codes recorded within hospital admissions data, containing 1,024 distinct codes (at the four-character level) for 16 conditions. The GP data contains 4,545 distinct Read Codes at the five-character level.

3. Common Mental Disorders (CMD)

The John et al, 2016 validated algorithm will be used to identify CMD in GP data.[30,40,41] The algorithm has utilised a combination of diagnosis, treatment and symptoms Read Codes in identifying CMD. Individuals with CMD are identified as either having a historical diagnosis code, currently treated or, having a current diagnosis/current symptom code. There are 89 distinct diagnosis codes, 15 symptom codes and 601 treatment codes.

4. Elixhauser Comorbidity Index

The Quan et al (2005) Elixhauser ICD-10 code list will be utilised for inpatient diagnosis and the Metcalfe et al (2019) Elixhauser Read Code list will be utilised for primary care recorded diagnosis.[33,34]

For peer review only

The ICD-10 codes have been taken from the pool of diagnosis codes recorded within hospital admissions data and contains 1,404 distinct codes (at the four-character level) for 30 conditions. The General practice data contains 6,074 distinct Read codes at the five-character level.

5. **Global Burden of Disease (GBD) Study**

The GBD 2019 ICD-10 codes will be used to identify 130 health conditions in secondary healthcare data. There are 3,497 distinct ICD-10 codes at the three and four-character level.[38]

6. **Quality Outcome Framework (QOF)**

The QOF conditions business rule V38 will be used to identify 18 health conditions in primary care data.[35] The 18 conditions are asthma, atrial fibrillation, obesity, coronary heart disease, chronic obstructive pulmonary disease, cancer, chronic kidney disease, dementia, depression, diabetes, epilepsy, heart failure, hypertension, learning difficulties, peripheral arterial disease, rheumatoid arthritis, serious mental illness and stroke. There are 2,275 distinct Read Codes available at the five-character level for the 18 QOF conditions.

Statistical analysis

The WMC provides an accessible research ready data asset to further understanding of multi-morbidity through the use of bio-statistical and machine learning approaches. Our collaborative team will work across a number of projects to develop and evaluate statistical and machine learning algorithms to address the following broad analytical challenges:

- What is the prevalence of multi-morbidity in the WMC, and how does prevalence of multi-morbidity change over time?
- What are common clusters of multi-morbidity in the WMC, and how do they correspond to or differ from, common clusters of multi-morbidity identified in other datasets?
- Which clusters of multi-morbidity occur less frequently than one would expect based on the prevalence of their constituent conditions?
- How does multi-morbidity develop across the life course (i.e. trajectories)?
- What are the biological, psychological, and social determinants of different clusters and trajectories of multi-morbidity?
- Which clusters and trajectories of multi-morbidity are associated with poor health outcomes?
- Which clusters and trajectories of multi-morbidity are associated with high service utilisation?
- Does multi-morbidity in specific groups (e.g. patients with musculoskeletal conditions) differ from multi-morbidity in general?

The overarching aim is to evaluate and provide new multi-morbidity phenotypes and algorithms to the NHS and research communities to support prevention, healthcare planning and the management of individuals with multi-morbidity.

For peer review only

We will draw upon both methods from statistics (e.g. regression analysis, longitudinal mixed models, multiple correspondence analysis, factor analysis [43], multi-state models, and latent class analysis) and machine learning (e.g. k-means clustering, semantic similarity clustering, market basket analysis, network models [44], and deep learning). We will use resampling methods to assess the stability of identified multi-morbidity clusters, and develop visualisation techniques to summarise multi-morbidity clusters and their associations with risk factors and outcomes.

Analyses will be coded in R, WinBUGS, and Python and made available to WMC users via a Git library to maximise transparency and reproducibility.[45]

Patient and public involvement

The proposal to develop WMC was submitted to the independent Information Governance Review Panel (IGRP) that includes members of the public (IGRP Project: 0911). We worked with this group to refine the study protocol. The scientific steering group includes two members of the public who have contributed to this paper. The HDR UK National Implementation Project Multi-morbidity Resource has a work package on PPI with a panel drawn from across the UK meeting to discuss the research work and feed into the research and dissemination plans.

Ethics and dissemination

The use of de-identified data in SAIL complies with National Research Ethics Service (NRES) guidance.[46] Applications to use data held within the SAIL Databank, an ISO: 27001 and UKSA DEA accredited TRE, must first be approved by the independent Information Governance Review Panel (IGRP). This panel contains individuals with expertise in data governance and protection, including the Chair of the Wales NRES Committee, Caldicott Guardians, and members of the public. WMC was approved by IGRP on 26th June 2019.

Findings from this study will be disseminated widely through a variety of routes, including to health policy and NHS leads across UK, the Academy of Medical Sciences and the Royal Colleges, as well as traditional scientific outlets. The team includes NHS clinicians and informaticians to allow for early NHS adoption of useful findings. Members of the public embedded in the team will create plain English summaries and lead at public facing meetings.

Contributors

Conceptualisation of study JL, AA, UA, GH, CM, DOR, RAL; data curation and analysis JL; original draft writing JL, review and editing of manuscript JL, AA, UA, GH, AAL, RB, JR, AW, RF, CM, CD, JPR, NP, CH, SD, RO, KRA, AJ, DOR, SR, MH, CPG, JD, CD,LC, JG, JC, AJB, RAL

For peer review only

Acknowledgements

This study makes use of anonymised data held in the Secure Anonymised Information Linkage (SAIL) Databank. We would like to acknowledge all the data providers and people who make anonymised data available for research. The authors would like to extend their gratitude and acknowledgement to the NHS, the SAIL Consumer panel as well as the IGRP who approved this project.

Funding

Two UK-wide collaborative efforts have been formed to address several of the AMS report priorities: *Measuring and Understanding Multi-morbidity using Routine Data in the UK – (MUrMuRUK)* and *Application of machine learning to discover new multi-morbidity phenotypes associated with poorer outcomes (WMML).*

This work was supported by Health Data Research UK (HDR-9006; CFC0110) and the Medical Research Council (MR/S027750/1). Health Data Research UK is funded by: UK Medical Research Council; Engineering and Physical Sciences Research Council; Economic and Social Research Council; National Institute for Health Research (England); Chief Scientist Office of the Scottish Government Health and Social Care Directorates; Health and Social Care Research and Development Division (Welsh Government); Public Health Agency (Northern Ireland); British Heart Foundation; and Wellcome Trust.

Disclaimer

The views and opinions expressed therein are those of the authors and do not necessarily reflect those of the funding agencies, NHS organisations or Welsh Government.

Competing interests

None declared.

For peer review only

References

1. The Academy of Medical Sciences. Multimorbidity: a priority for global health research, April 2018. https://acmedsci.ac.uk/file-download/82222577

2. WHO. World Health Organization. The Challenges of a changing world. The World Health Report 2008—primary Health Care (Now More Than Ever). 2008 https://journals.plos.org/plosone/article/file?id=10.1371/journal.pone.0237186&type=printable

3. Barnett K, Mercer SW, Norbury M, Watt G, Wyke S, Guthrie B. Epidemiology of multimorbidity and implications for health care, research, and medical education: a cross-sectional study. The Lancet. 2012;380(9836):37–43.
4. Cassell A, Edwards D, Harshfield A, Rhodes K, Brimicombe J, Payne R, et al. The epidemiology of multimorbidity in primary care: a retrospective cohort study. British Journal of General Practice. 2018;68(669).
5. Nunes BP, Flores TR, Mielke GI, Thumé E, Facchini LA. Multimorbidity and mortality in older adults: A systematic review and meta-analysis. *Arch Gerontol Geriatr*. 2016;67:130-138. Doi:10.1016/j.archger.2016.07.008
6. Hall, M., Dondo, T. B., Yan, A. T., Mamas, M. A., Timmis, A. D., Deanfield, J. E., ... & Gale, C. P. (2018). Multimorbidity and survival for patients with acute myocardial infarction in England and Wales: Latent class analysis of a nationwide population-based cohort. *PLoS medicine*, *15*(3), e1002501.
7. Kingston A, Robinson L, Booth H, et al. Projections of multi-morbidity in the older population in England to 2035: estimates from the Population Ageing and Care Simulation (PACSim) model. Age Ageing. 2018. doi: 10.1093/ageing/afx201
8. Diederichs C, Berger K, Bartels D. The measurement of multiple chronic diseases—a systematic review on existing multimorbidity indices. J Gerontol A Biol Sci Med Sci 2011; 66: 301–11.
9. Ford JC, Ford JA. Multimorbidity: will it stand the test of time? Age Ageing. 2018;47(1):6–8.
10. Vos T, Flaxman AD, Naghavi M, Lozano R, Michaud C, Ezatti M, et al. Years lived with disability (YLDs) for 1160 sequelae of 289 diseases and injuries 1990—2010: a systematic analysis for the Global Burden of Disease Study 2010. The Lancet 2012;380(9859):2163 – 2196. (15 December 2012). doi:10.1016/S0140-6736(12)61729-2.
11. Steel N, Ford J, Newton J, Davis A, Vos T, Naghavi M et al. Mortality, causes of death, years of life lost, years lived with a disability, and disability-adjusted life years in the countries of the UK and 150 English Local Authority areas 1990-2016: a systematic analysis for the Global Burden of Disease Study 2016. Accepted Lancet 02/09/18.
12. Bauermeister S, Orton C, Thompson S. et al. The Dementias Platform UK (DPUK) Data Portal. European Journal of Epidemiology 2020;35:601-611. https://doi.org/10.1007/s10654-020-00633-4
13. Lyons RA, Jones KH, John G, Brooks CJ, Verplancke JP, Ford DV, Brown G, Leake K. The SAIL databank: linking multiple health and social care datasets. BMC Med Inform Decis Mak. 2009 Jan 16;9:3. http://www.biomedcentral.com/1472-6947/9/3
14. Ford DV, Jones KH, Verplancke JP, Lyons RA, John G, Brown G, Brooks CJ, Thompson S, Bodger O, Couch T, Leake K. The SAIL Databank: building a national architecture for e-health research and evaluation. BMC Health Services Research 2009;9:157 doi:10.1186/1472-6963-9-157
15. Lyons RA**,** Ford DV, Moore L, Rodgers SE. Using data linkage to measure the population health impact of non-healthcare interventions. The Lancet 2014;383:1517-1518.
16. Lyons RA, Turner S, Lyons J, Walters A, Snooks HA, Greenacre J, Humphreys C, Jones SJ. All Wales Injury Surveillance System revised: development of a population based system to evaluate single and multi-level interventions. Inj Prev 2015;0:1-6. Published Online First: [09/12/15] doi:10.1136/injuryprev-2015-041814
17. Snooks HA, Anthony R, Chatters R, Dale J, Fothergill R, Gaze S, et al. Support and Assessment for Fall Emergency Referrals (SAFER) 2: a cluster randomised trial and systematic review of clinical effectiveness and cost-effectiveness of new protocols for emergency ambulance paramedics to

assess older people following a fall with referral to community-based care when appropriate. Health Technology Assessment. 2017;21(13):1–218.
18. Hollingsworth JP, Rodgers SE, Akbari A, Mizen A, Berridge DM, Clegg A, Walters A, Lyons RA, Williams N. A study protocol for investigating the impact of community home modification services on hospital utilisation for fall injuries: a controlled longitudinal study using data linkage. BMJ Open 2018:8:e026290. doi: 10.1136/bmjopen-2018-026290.
19. Mizen A, Song J, Fry R, Akbari A, Berridge D, Johnson R, Rebecca Lovell R, Lyons RA, Mark Nieuwenhuijsen M, Gareth Stratton G, Wheeler BW, White J, White M, Rodgers S. Longitudinal access and exposure to green-blue spaces and individual-level mental health and wellbeing: A study programme protocol for a longitudinal, population-wide record-linked natural experiment. BMJ Open 2019;9:e027289. Doi:10.1136/bmjopen-2018-027289
20. Szakmany S, Walters AM, Pugh R, Battle C, Berridge DM, Lyons RA. Risk factors for 1-year mortality and healthcare utilisation patterns in critical care survivors: a retrospective, observational, population-based data-linkage study. Crit Care Med 2019;47:15-22. doi: 10.1097/CCM.0000000000003424
21. Rodgers SE, Bailey R, Johnson R, Berridge DM, Poortinga W, Lannon S, Smith R, Lyons RA. Emergency hospital admissions associated with a non randomised housing intervention meeting national housing quality standards: a longitudinal data linkage study. J Epidemiol Community Health 2018;0:1-8 doi: 10.1136/jech-2017-210370
22. Paranjothy S, Evans A, Bandyopathy A, Fone D, Schofield B, John A, Bellis MA, Lyons RA, Farewell D, Long SL. Risk of emergency hospital admissions associated with mental disorders and alcohol misuse in the household: an electronic birth cohort study. Lancet Public Health 2018;3;e279-288. https://doi.org/10.1016/S2468-2667(18)30069-0 .
23. Schnier C, Wilkinson T, Akbari A, Orton C, Sleegers K, Gallacher J, Lyons RA, Sudlow CLM, on behalf of Dementias Platform UK. Cohort profile: The Secure Anonymised Information Linkage Databank Dementia e-cohort (SAIL-DeC). IJPDS 2020 (published 25/01/20) https://doi.org/10.23889/ijpds.v5i1.1121.
24. Discontinuities in results for health-related lifestyle and general health between the Welsh Health Survey and National Survey for Wales. Available: https://gov.wales/sites/default/files/statistics-and-research/2019-02/discontinuities-results-health-related-lifestyle-general-health-between-welsh-health-survey-national-survey-wales-2018.pdf [Accessed 24 August 2020].
25. Rodgers SE, Lyons RA, Dsilva R, Jones KH, Brooks CJ, Ford DV, et al. Residential Anonymous Linking Fields (RALFs): a novel information infrastructure to study the interaction between the environment and individuals' health. Journal of Public Health. 2009;31(4):582–8.
26. Welsh Index Multiple Deprivation Index. Available: https://gov.wales/welsh-index-multiple-deprivation-index-guidance [Accessed 9 April 2020].
27. 2011 rural/urban classifications. Available: https://www.ons.gov.uk/methodology/geography/geographicalproducts/ruralurbanclassifications/2011ruralurbanclassification [Accessed 9 April 2020].
28. Thayer D, Rees A, Kennedy J, Collins H, Harris D, Halcox J, et al. Measuring follow-up time in routinely-collected health datasets: Challenges and solutions. Plos One. 2020;15(2).
29. Nils Gehlenborg (2019). UpSetR: A More Scalable Alternative to Venn and Euler Diagrams for Visualizing Intersecting Sets. R package version 1.4.0. https://CRAN.R-project.org/package=UpSetR
30. John, A., McGregor, J., Fone, D. *et al.* Case-finding for common mental disorders of anxiety and depression in primary care: an external validation of routinely collected data. *BMC Med Inform Decis Mak* **16,** 35 (2016). https://doi.org/10.1186/s12911-016-0274-7
31. Kuan V, Denaxas S, Gonzalez-Izquierdo A, Direk K, Bhatti O, Husain S, et al. A chronological map of 308 physical and mental health conditions from 4 million individuals in the English National Health Service. The Lancet Digital Health. 2019;1(2).
32. Bottle A, Aylin P. Comorbidity scores for administrative data benefited from adaptation to local coding and diagnostic practices. Journal of Clinical Epidemiology. 2011;64(12):1426–33.
33. Metcalfe D, Masters J, Delmestri A, Judge A, Perry D, Zogg C, et al. Coding algorithms for defining Charlson and Elixhauser co-morbidities in Read-coded databases. BMC Medical Research Methodology. 2019;19(1).

For peer review only - http://bmjopen.bmj.com/site/about/guidelines.xhtml

34. Quan H, Sundararajan V, Halfon P, Fong A, Burnand B, Luthi J-C, et al. Coding Algorithms for Defining Comorbidities in ICD-9-CM and ICD-10 Administrative Data. Medical Care. 2005;43(11):1130–9.
35. Quality and Outcomes Framework (QOF) business rules v 38 2017-2018 October code release. Available: https://digital.nhs.uk/data-and-information/data-collections-and-data-sets/data-collections/quality-and-outcomes-framework-qof/quality-and-outcome-framework-qof-business-rules/quality-and-outcomes-framework-qof-business-rules-v-38-2017-2018-october-code-release [Accessed 21st August 2019]
36. Charlson ME, Pompei P, Ales KL, MacKenzie CR; A new method of classifying prognostic comorbidity in longitudinal studies: development and validation; J Chron Dis, 1987, 40: 373-383.
37. Elixhauser A, Steiner C, Harris R, Coffey RM; Comorbidity Measures For Use With Administrative Data; Medical Care, 1998, 36:8-27
38. Global Burden of Disease Collaborative Network. Global Burden of Disease Study 2017 (GBD 2017) Causes of Death and Nonfatal Causes Mapped to ICD Codes. Seattle, United States of America: Institute for Health Metrics and Evaluation (IHME), 2018. Available at: http://ghdx.healthdata.org/record/ihme-data/gbd-2017-cause-icd-code-mappings[Accessed 01 June 2020]
39. J Am Med Inform Assoc. 2019 Dec 1;26(12):1545-1559. doi: 10.1093/jamia/ocz105.
40. John A, DelPozo-Banos M, Gunnell D, Dennis M, Scourfield J, Ford DV, et al. Contacts with primary and secondary healthcare prior to suicide: case-control whole-population-based study using person-level linked routine data in Wales, UK, 2000-2017. Br J Psychiatry. 2020;1–8.
41. Ware JE Jr, Gandek B. Overview of the SF-36 Health Survey and the International Quality of Life Assessment (IQOLA) Project. J Clin Epidemiol. 1998;51(11):903–12.
42. Legally unsharable clinical codes - NHS Digital - Citizen Space [Internet]. Citizenspace.com. [cited 2020 Nov 9]. Available from: https://nhs-digital.citizenspace.com/standards-assurance/legally-unsharable-clinical-codes
43. Pages J. Multiple factor analysis by example using R [Internet]. Philadelphia, PA: Chapman & Hall/CRC; 2014. Available from: http://dx.doi.org/10.1201/b17700
44. Marx P, Antal P, Bolgar B, Bagdy G, Deakin B, Juhasz G. Comorbidities in the diseasome are more apparent than real: What Bayesian filtering reveals about the comorbidities of depression. PLoS Comput Biol. 2017 Jun 23;13(6):e1005487.
45. Lunn, D.J., Thomas, A., Best, N., and Spiegelhalter, D. (2000) WinBUGS — a Bayesian modelling framework: concepts, structure, and extensibility. *Statistics and Computing*, **10**:325–337.
46. Jones KH et al.The Secure Anonymous Information Linkage (SAIL) Gateway: a case study describing a remote access system for health-related research and evaluation. Journal of Biomedical Informatics 01/2014; DOI:10.1016/j.jbi.2014.01.003

For peer review only

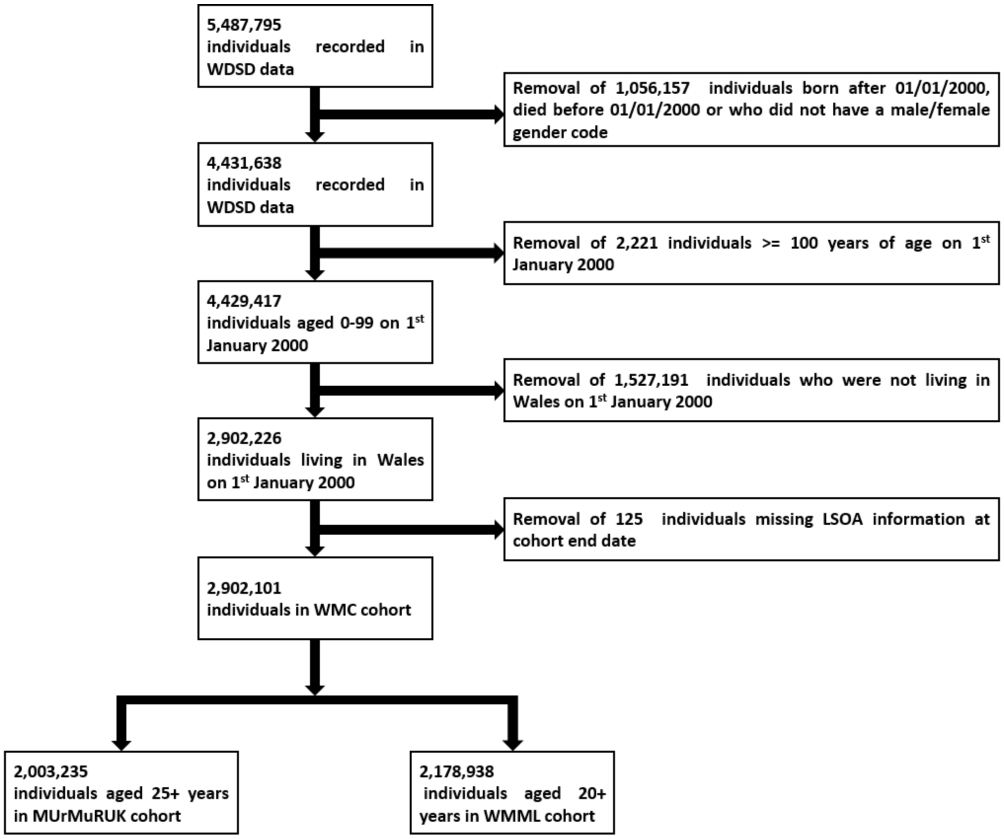

Figure 2: WMC flow diagram, based on inclusion criteria.

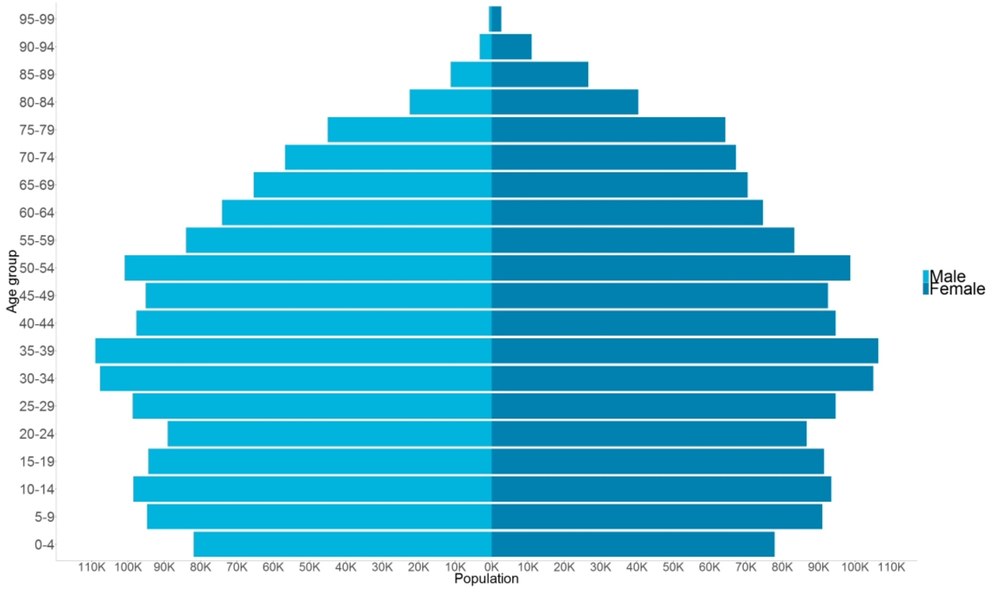

Figure 3: WMC pyramid for age (years) at cohort inception.

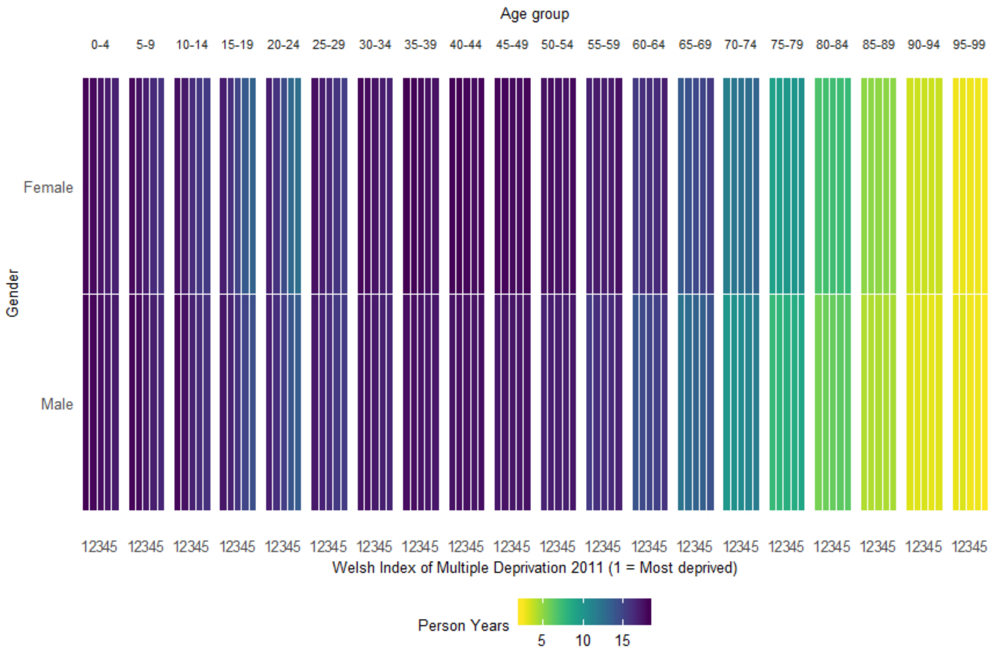

Figure 4: Heatmap of person years of WMC follow up, by age group, sex and area level deprivation at cohort inception.

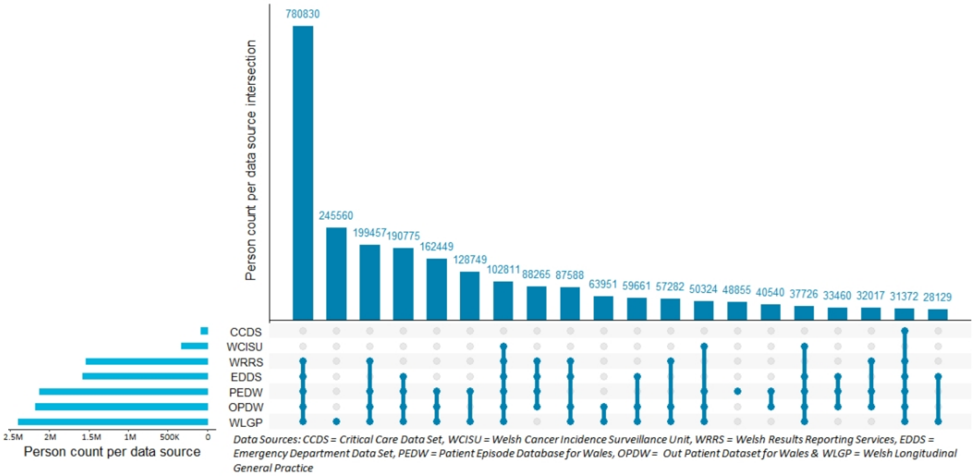

Figure 5: Number of WMC individuals utilising healthcare services recorded in multi-source data sources, 20 most common combinations presented.

Appendix

Table A1: WMC participants categorised by age group and sex at cohort start

Age group	Sex	Count	Percentage
00-04	Male	81,915	2.82
00-04	Female	77,873	2.68
05-09	Male	94,737	3.26
05-09	Female	90,940	3.13
10-14	Male	98,466	3.39
10-14	Female	93,447	3.22
15-19	Male	94,345	3.25
15-19	Female	91,440	3.15
20-24	Male	89,037	3.07
20-24	Female	86,666	2.99
25-29	Male	98,622	3.40
25-29	Female	94,592	3.26
30-34	Male	107,671	3.71
30-34	Female	104,986	3.62
35-39	Male	108,964	3.75
35-39	Female	106,312	3.66
40-44	Male	97,637	3.36
40-44	Female	94,599	3.26
45-49	Male	95,071	3.28
45-49	Female	92,478	3.19
50-54	Male	100,866	3.48
50-54	Female	98,606	3.40
55-59	Male	83,949	2.89
55-59	Female	83,210	2.87
60-64	Male	74,115	2.55
60-64	Female	74,591	2.57
65-69	Male	65,354	2.25
65-69	Female	70,389	2.43
70-74	Male	56,746	1.96
70-74	Female	67,227	2.32
75-79	Male	45,027	1.55
75-79	Female	64,274	2.21
80-84	Male	22,441	0.77
80-84	Female	40,344	1.39
85-89	Male	11,184	0.39
85-89	Female	26,540	0.91
90-94	Male	3,208	0.11
90-94	Female	10,951	0.38
95-99	Male	633	0.02
95-99	Female	2,648	0.09

For peer review only

Table A2: WMC average person years of follow up, categorised by age group, sex and WIMD 2011 at cohort start

Age group	Sex	WIMD 2011 quintiles	Average Pys
00-04	Male	1	18.16
00-04	Male	2	17.93
00-04	Male	3	17.63
00-04	Male	4	17.28
00-04	Male	5	17.11
05-09	Male	1	18.06
05-09	Male	2	17.81
05-09	Male	3	17.26
05-09	Male	4	16.91
05-09	Male	5	16.50
10-14	Male	1	17.93
10-14	Male	2	17.46
10-14	Male	3	16.76
10-14	Male	4	16.24
10-14	Male	5	15.98
15-19	Male	1	17.30
15-19	Male	2	16.73
15-19	Male	3	15.75
15-19	Male	4	14.89
15-19	Male	5	14.34
20-24	Male	1	16.96
20-24	Male	2	16.04
20-24	Male	3	15.29
20-24	Male	4	14.03
20-24	Male	5	13.59
25-29	Male	1	17.18
25-29	Male	2	16.71
25-29	Male	3	16.22
25-29	Male	4	15.57
25-29	Male	5	15.47
30-34	Male	1	17.44
30-34	Male	2	17.41
30-34	Male	3	17.11
30-34	Male	4	16.82
30-34	Male	5	16.60
35-39	Male	1	17.66
35-39	Male	2	17.63
35-39	Male	3	17.41
35-39	Male	4	17.27

For peer review only

35-39	Male	5	17.22
40-44	Male	1	17.50
40-44	Male	2	17.63
40-44	Male	3	17.55
40-44	Male	4	17.40
40-44	Male	5	17.57
45-49	Male	1	17.23
45-49	Male	2	17.39
45-49	Male	3	17.28
45-49	Male	4	17.24
45-49	Male	5	17.48
50-54	Male	1	16.68
50-54	Male	2	16.96
50-54	Male	3	16.89
50-54	Male	4	16.91
50-54	Male	5	17.30
55-59	Male	1	15.46
55-59	Male	2	16.03
55-59	Male	3	16.20
55-59	Male	4	16.31
55-59	Male	5	16.78
60-64	Male	1	14.07
60-64	Male	2	14.64
60-64	Male	3	14.96
60-64	Male	4	15.19
60-64	Male	5	15.80
65-69	Male	1	12.14
65-69	Male	2	12.70
65-69	Male	3	13.24
65-69	Male	4	13.46
65-69	Male	5	14.21
70-74	Male	1	9.73
70-74	Male	2	10.23
70-74	Male	3	10.59
70-74	Male	4	11.02
70-74	Male	5	11.58
75-79	Male	1	7.46
75-79	Male	2	7.73
75-79	Male	3	8.14
75-79	Male	4	8.29
75-79	Male	5	8.79
80-84	Male	1	5.55
80-84	Male	2	5.82
80-84	Male	3	6.02

For peer review only

80-84	Male	4	6.24
80-84	Male	5	6.30
85-89	Male	1	4.20
85-89	Male	2	4.18
85-89	Male	3	4.28
85-89	Male	4	4.27
85-89	Male	5	4.41
90-94	Male	1	3.09
90-94	Male	2	3.07
90-94	Male	3	3.22
90-94	Male	4	2.95
90-94	Male	5	3.13
95-99	Male	1	2.87
95-99	Male	2	3.19
95-99	Male	3	2.64
95-99	Male	4	2.77
95-99	Male	5	2.32
00-04	Female	1	18.03
00-04	Female	2	17.82
00-04	Female	3	17.37
00-04	Female	4	16.99
00-04	Female	5	16.73
05-09	Female	1	17.84
05-09	Female	2	17.44
05-09	Female	3	16.89
05-09	Female	4	16.27
05-09	Female	5	15.92
10-14	Female	1	17.57
10-14	Female	2	17.09
10-14	Female	3	16.20
10-14	Female	4	15.60
10-14	Female	5	15.51
15-19	Female	1	16.93
15-19	Female	2	16.08
15-19	Female	3	14.88
15-19	Female	4	13.68
15-19	Female	5	13.21
20-24	Female	1	16.94
20-24	Female	2	15.81
20-24	Female	3	14.61
20-24	Female	4	12.89
20-24	Female	5	12.48
25-29	Female	1	17.53
25-29	Female	2	16.99

25-29	Female	3	16.47
25-29	Female	4	15.77
25-29	Female	5	15.34
30-34	Female	1	17.94
30-34	Female	2	17.77
30-34	Female	3	17.39
30-34	Female	4	16.94
30-34	Female	5	16.77
35-39	Female	1	18.18
35-39	Female	2	18.12
35-39	Female	3	17.78
35-39	Female	4	17.47
35-39	Female	5	17.49
40-44	Female	1	18.11
40-44	Female	2	18.16
40-44	Female	3	17.91
40-44	Female	4	17.71
40-44	Female	5	17.92
45-49	Female	1	17.92
45-49	Female	2	17.93
45-49	Female	3	17.82
45-49	Female	4	17.66
45-49	Female	5	17.97
50-54	Female	1	17.49
50-54	Female	2	17.69
50-54	Female	3	17.49
50-54	Female	4	17.44
50-54	Female	5	17.87
55-59	Female	1	16.79
55-59	Female	2	17.09
55-59	Female	3	17.00
55-59	Female	4	17.06
55-59	Female	5	17.54
60-64	Female	1	15.53
60-64	Female	2	16.04
60-64	Female	3	16.23
60-64	Female	4	16.28
60-64	Female	5	16.96
65-69	Female	1	13.76
65-69	Female	2	14.28
65-69	Female	3	14.70
65-69	Female	4	14.92
65-69	Female	5	15.52
70-74	Female	1	11.43

For peer review only

70-74	Female	2	11.93
70-74	Female	3	12.27
70-74	Female	4	12.57
70-74	Female	5	13.12
75-79	Female	1	9.06
75-79	Female	2	9.35
75-79	Female	3	9.70
75-79	Female	4	9.82
75-79	Female	5	10.25
80-84	Female	1	6.78
80-84	Female	2	7.07
80-84	Female	3	7.19
80-84	Female	4	7.26
80-84	Female	5	7.50
85-89	Female	1	4.98
85-89	Female	2	5.02
85-89	Female	3	4.97
85-89	Female	4	5.07
85-89	Female	5	5.07
90-94	Female	1	3.45
90-94	Female	2	3.53
90-94	Female	3	3.61
90-94	Female	4	3.55
90-94	Female	5	3.66
95-99	Female	1	2.66
95-99	Female	2	2.87
95-99	Female	3	2.70
95-99	Female	4	2.75
95-99	Female	5	2.48

For peer review only

BMJ Open

Protocol for the development of the Wales Multi-morbidity e-Cohort (WMC): data sources and methods to construct a population-based research platform to investigate multi-morbidity.

Journal:	*BMJ Open*
Manuscript ID	bmjopen-2020-047101.R1
Article Type:	Protocol
Date Submitted by the Author:	22-Dec-2020

Complete List of Authors:	Lyons, Jane; Swansea University Medical School,<br>Akbari, Ashley; Swansea University Medical School,<br>Agrawal, Utkarsh; University of St Andrews, School of Medicine<br>Harper, Gill; Queen Mary University of London<br>Azcoaga-Lorenzo, Amaya; University of Saint Andrews School of Medicine, Division of Population and Behavioural Sciences<br>Bailey, Rowena; Swansea University Medical School, Population Data Science<br>Rafferty, James; Swansea University Medical School<br>Watkins, Alan; Swansea University, College of Medicine<br>Fry, Richard; Swansea University, Medical School<br>McCowan, Colin ; University of St Andrews<br>Dezateux, Carol; Queen Mary University of London, Centre for Primary Care and Public Health<br>Robson, John; Barts and The London School of Medicine and Dentistry, Queen Mary University of London, Centre for Primary Care and Public Health<br>Peek, N; Health e-Research Centre, Institute of Population Health, University of Manchester<br>Holmes, Chris; Oxford University<br>Denaxas, S; University College London<br>Owen, R; University of Leicester<br>Abrams, Keith; University of Leicester, Biostatistics Research Group, Department of Health Sciences<br>John, Ann; Swansea University<br>OReilly, Dermot; Queens University Belfast, Epidemiology and Public Health<br>Richardson , Sylvia; MRC Biostatistics Unit, Department of Epidemiology and Public Health<br>Hall, Marlous ; Leeds<br>Gale, Chris; University of Leeds<br>Davies, Jan; Swansea University,<br>Davies, Chris; Swansea University,<br>Cross, Lynsey; Swansea University Medical School, Population Data Science<br>Gallacher, John; Oxford University<br>Chess, James; Swansea Bay University Health Board, Renal Unit<br>Brookes, Anthony; University of Leicester

	Lyons, Ronan; Swansea University, Swansea Clinical School
**Primary Subject Heading**:	Epidemiology
Secondary Subject Heading:	Public health, Research methods, Health informatics
Keywords:	PUBLIC HEALTH, EPIDEMIOLOGY, Health policy < HEALTH SERVICES ADMINISTRATION & MANAGEMENT, PRIMARY CARE, GERIATRIC MEDICINE

SCHOLARONE™ Manuscripts

I, the Submitting Author has the right to grant and does grant on behalf of all authors of the Work (as defined in the below author licence), an exclusive licence and/or a non-exclusive licence for contributions from authors who are: i) UK Crown employees; ii) where BMJ has agreed a CC-BY licence shall apply, and/or iii) in accordance with the terms applicable for US Federal Government officers or employees acting as part of their official duties; on a worldwide, perpetual, irrevocable, royalty-free basis to BMJ Publishing Group Ltd ("BMJ") its licensees and where the relevant Journal is co-owned by BMJ to the co-owners of the Journal, to publish the Work in this journal and any other BMJ products and to exploit all rights, as set out in our licence.

The Submitting Author accepts and understands that any supply made under these terms is made by BMJ to the Submitting Author unless you are acting as an employee on behalf of your employer or a postgraduate student of an affiliated institution which is paying any applicable article publishing charge ("APC") for Open Access articles. Where the Submitting Author wishes to make the Work available on an Open Access basis (and intends to pay the relevant APC), the terms of reuse of such Open Access shall be governed by a Creative Commons licence – details of these licences and which Creative Commons licence will apply to this Work are set out in our licence referred to above.

Other than as permitted in any relevant BMJ Author's Self Archiving Policies, I confirm this Work has not been accepted for publication elsewhere, is not being considered for publication elsewhere and does not duplicate material already published. I confirm all authors consent to publication of this Work and authorise the granting of this licence.

For peer review only

Protocol for the development of the Wales Multi-morbidity e-Cohort (WMC): data sources and methods to construct a population-based research platform to investigate multi-morbidity.

Jane Lyons, Data Science Building, Population Data Science, Swansea University Medical School, Singleton Park, Swansea, SA2 8PP, UK, J.Lyons@Swansea.ac.uk, 01792 513028 (corresponding author)

Ashley Akbari, Swansea University, Swansea, UK

Dr Utkarsh Agrawal, University of St Andrews, St Andrews, UK

Dr Gill Harper, Queen Mary University of London, London, UK

Dr Amaya Azcoaga-Lorenzo, University of St Andrews, St Andrews, UK

Rowena Bailey, Swansea University, Swansea, UK

Dr James Rafferty, Swansea University, Swansea, UK

Professor Alan Watkins, Swansea University, Swansea, UK

Dr Richard Fry, Swansea University, Swansea, UK

Professor Colin McCowan, University of St Andrews, St Andrews, UK

Professor Carol Dezateux, Queen Mary University of London, London, UK

Dr John P Robson, Queen Mary University of London, London, UK

Professor Niels Peek, University of Manchester, Manchester, UK

Professor Chris Holmes, University of Oxford, Oxford, UK

Professor Spiros Denaxas, University College London, London, UK

Dr Rhiannon Owen, University of Leicester, Leicester, UK

Professor Keith R Abrams, University of Leicester, Leicester, UK

Professor Ann John, Swansea University, Swansea, UK

Professor Dermot O'Reilly, Queens University, Belfast, UK

Professor Sylvia Richardson, Cambridge University, Cambridge, UK

Dr Marlous Hall, University of Leeds, Leeds, UK

Professor Chris P Gale, University of Leeds, Leeds UK

Jan Davies, Member of public, Swansea, UK

Chris Davies, Member of public, Swansea, UK

Lynsey Cross, Swansea University, Swansea, UK

Professor John Gallacher, Oxford University, Oxford, UK

Dr James Chess, Swansea Bay University Health Board, Swansea, UK

Professor Anthony J Brookes, University of Leicester, Leicester, UK

Professor Ronan A Lyons, Swansea University, Swansea, UK

Keywords: Multi-morbidity, data linkage, epidemiology, public health, evaluation

For peer review only

Word count: 2785/4000

ABSTRACT

Introduction

Multi-morbidity is widely recognised as the presence of two or more concurrent long-term conditions, but remains a poorly understood global issue despite increasing in prevalence.

We have created the Wales Multi-morbidity e-Cohort (WMC) to provide an accessible research ready data asset to further the understanding of multi-morbidity. Our objectives are to create a platform to support research which would help to understand prevalence, trajectories and determinants in multi-morbidity, characterise clusters that lead to highest burden on individuals and healthcare services, and evaluate and provide new multi-morbidity phenotypes and algorithms to the NHS and research communities to support prevention, healthcare planning and the management of individuals with multi-morbidity.

Methods and analysis

The WMC has been created and derived from multi-sourced demographic, administrative and electronic health record (EHR) data relating to the Welsh population in the Secure Anonymised Information Linkage (SAIL) Databank. The WMC consists of 2.9 million people alive and living in Wales on the 1st January 2000 with follow up until 31st December 2019, Welsh residency break or death. Published comorbidity indices and phenotype code lists will be used to measure and conceptualise multi-morbidity.

Study outcomes will include: a) a description of multi-morbidity using published data phenotype algorithms/ontologies, b) investigation of the associations between baseline demographic factors and multi-morbidity c) identification of temporal trajectories of clusters of conditions and multi-morbidity, d) investigation of multi-morbidity clusters with poor outcomes such as mortality and high healthcare service utilisation.

Ethics and dissemination

The SAIL Databank independent Information Governance Review Panel (IGRP) has approved this study (SAIL Project: 0911). Study findings will be presented to policy groups, public meetings, national and international conferences, and published in peer-reviewed journals.

For peer review only

Strengths and limitations of this study

- Creation and access to a multi-sourced population based, deeply phenotyped e-cohort.
- Future use of this resource removes need for data management and cleaning of source data, accelerating research and which could also support efforts for reproducibility of results.
- Variety of individual and household level data on demography, health status, health care utilisation, both primary and secondary healthcare, and mortality to support a wide range of analytical approaches to addressing scientific questions.
- Input from multiple disciplines and institutions from across all four nations of the United Kingdom to help understand, measure and address multi-morbidity.
- Routine data does not capture data on some important aspects, such as quality of life.

For peer review only

INTRODUCTION

Multi-morbidity is defined by the UK's Academy of Medical Sciences (AMS) and the World Health Organisation as the presence of two or more concurrent long-term conditions, which is a global and growing phenomenon.[1,2] Multi-morbidity is more prevalent in older individuals and associated with high healthcare utilisation and mortality, but with large numbers of patients of all age suffering from multi-morbidity.[3-6] With an aging population, it is estimated that two in three people in England aged 65 years or over will experience multi-morbidity by 2035 and nearly one fifth will have complex multi-morbidity (4 or more conditions).[7]

Much of what is known about multi-morbidity is based on a limited and fragmented knowledge base, largely derived from studies of older people in high-income countries or hospital populations.[1,8] The 2018 AMS report concluded that multi-morbidity is an unhelpful term implying random assortment of disease when it often refers to clusters of specific diseases. Once identified, these disease clusters can be addressed specifically through research, healthcare policy development and service delivery.[1,9] The identification of previously unrecognised disease clusters may also provide biological and clinical insights into their aetiology, prevention and treatment. The AMS report identified specific research gaps and proposed a list of priorities (Textbox 1). Several can be addressed through a combination of health data science, epidemiology and statistics, and by exploiting the potential from creating deeply phenotyped cohorts from population and clinical data sources.

Textbox 1: The Academy of Medical Sciences identified research gaps.

- The scale and nature of multi-morbidity and how it is changing over time.
- Which clusters of conditions cause the biggest problems for patients.
- The causes of the most common clusters including links with sex, ethnicity, income and lifestyle.
- The best ways to prevent the patients developing multi-morbidity, and whether this requires different approaches to just preventing individual conditions.
- How doctors can increase the benefits and reduce the risks of treatment for patients with multi-morbidity.
- How to organise healthcare systems to deal with multi-morbidity more effectively and how best to use digital technology in caring for patients.

Responding to this agenda, we created a privacy protecting total population electronic cohort - the Wales Multi-morbidity e-Cohort (WMC) - as a platform to study these issues in depth, collaborating with scientists

For peer review

from many different institutions and disciplines, clinicians, and members of the public from across the UK to create a broader team science approach.

The objectives of this work are to understand prevalence, trajectories and determinants of multi-morbidity, and identify clusters causing the greatest health care burden. The WMC will also contribute data on incidence, prevalence and burden to the Global Burden of Diseases Study,[10,11] and provide new multi-morbidity phenotypes to e-cohorts with local participants, and phenotyping algorithms to many e-cohorts that utilise routine data.[12]

We expect that findings from these analyses will provide evidence to health policy leads in order to support prevention and the complex healthcare planning and management of multi-morbid individuals. Members of the public are embedded in the research team to ensure the resource focuses on issues of concern to the public.

This paper describes the creation of the WMC and the statistical approaches that will be developed to support the diverse research objectives.

METHODS

The WMC was developed by linking multiple routinely collected population and clinical data sources on the population of Wales from 2000-2019. We used the privacy-protecting Secure Anonymised Information Linkage (SAIL) Databank, to contribute to the Health Data Research UK National Implementation Multi-morbidity Resource (HNIMR) project, and extended to 2020 for the MRC funded Welsh Multi-morbidity Machine Learning (WMML) project .[13,14] SAIL is one of the most comprehensive, privacy protecting, linked data Trusted Research Environments (TRE) in the UK. SAIL utilising data from many different sources and providing linkage at individual and household level.[15] It has supported many different study designs, including, large-scale community-based or clinical condition-based observational studies, disease surveillance, evaluation of natural experiments of environmental interventions, embedded trials, and the Dementias Platform UK.[16-23]

Cohort design and characteristics

WMC is a clearly defined complete population cohort. Cohort entry includes all residents in Wales, alive and living on the 1st January 2000. Cohort censorship was defined by the first date of migration out of Wales/residency break, death or the study endpoint on 31st December 2019 (Figure 1). Within these constraints, the cohort is designed to be without selection bias and to achieve complete follow-up. WMC also provides a fully generalisable population sample against which findings from more selected samples may be compared.

For peer review only

The WMC contains 2,902,101 individuals aged 0-99 at cohort start date with 46 million person years of follow up available (Table 1, Figures 2 & 3, Appendix Table A1 & A2). Individuals have a minimum of 1-day follow up (cohort end date = 2nd January 2000) and maximum of 20-years of follow up (cohort end date = 31st December 2019).

Table 1: WMC baseline demographics

WMC characteristics	n	%
Cohort size	2,902,101	100
Full coverage (01-01-2000 – 31-12-2019)	1,714,484	59.08
Residency break/Emigration	643,472	22.17
Mortality	544,145	18.75
Primary care data available	2,470,874	85.14
Care home residency at cohort end	97,006	3.34
Mean age in years (range) at cohort start	39 (0-99)	
Sex		
Female	1,472,113	50.60
Male	1,436,988	49.40
WIMD 2011 Quintile at cohort start		
1. Most Deprived	605,203	20.85
2	589,479	20.31
3	584,039	20.12
4	557,319	19.20
5. Least Deprived	566,061	19.51

The Heatmap in Figure 3 visualises the person years of follow up by age, sex and area level deprivation. The more years of follow up available the darker the colour. Age is calculated at the cohort start, therefore, younger individuals will have more years of available follow up compared to older individuals. On average, there are less person years of follow up available for the least deprived 15-24 year olds compared to their respective age group in other areas of Wales.

Data Sources

The WMC has utilised and combined anonymised health, social and environmental data held within the SAIL Databank (www.saildatabank.com).

For peer review only

The baseline characteristics for the WMC have been created using the Welsh Demographic Service Dataset (WDSD) and the Annual District Death Extract (ADDE) mortality registry data from the Office for National Statistics. The WDSD contains administrative information concerning the resident population of Wales that are registered to a Welsh General Practice, a free to use National Health Service (NHS) system at the point of primary care registration in the UK. The ADDE data contains information about the dates and causes of all deaths relating to residents in Wales, including those that died outside of Wales. SAIL holds GP data for approximately 80% of the population with coverage extending to all local authorities in Wales. The Welsh Longitudinal General Practice (WLGP) data will be used to identify the sub-population of individuals who are registered to a practice providing data to SAIL to identify which individuals have GP data present and avoid underestimation of conditions or severity of conditions not managed through hospital admission.

The Welsh Health Survey Dataset (WHSD) and the National Survey for Wales Dataset (NSWD) with data on wellbeing measures, social class, education, housing and wealth are available for 9,905 and 33,295 cohort participants respectively. [24]

Anonymised Linkage Fields

Linkage fields are used to anonymously link between data sources in the SAIL Databank and have been previously described elsewhere.[13,14,25] SAIL utilises a multiple encryption system in which a trusted third party, the National Health Service (NHS) Wales Informatics Service (NWIS), uniquely matches identities (NHS number, name, date of birth, and residential address/UPRN) and replaces these with unique identifiers. For individuals this is called an Anonymised Linkage Field (ALF) and Residential Anonymised Linkage Field (RALF) for pseudonymised residences before uploading data to SAIL.

Demographic Data

The cohort includes the following variables: Anonymised Linkage Field (ALF), age in years, sex, date of death, date of movement out of Wales, RALF at both cohort inception and cohort end and Care Home Anonymised Linkage Fields (CHALFs) at cohort end date. The CHALF was derived from a data extract from Care Inspectorate Wales in 2020 for all adult care home settings.[18] Geographical variables associated with the RALF and CHALF include Lower layer Super Output Area (LSOA) 2001 at cohort inception and LSOA 2011 at cohort end. These have been mapped to the Welsh Index of Multiple Deprivation (WIMD) version 2011 and 2019 respectively to derive socioeconomic deprivation quintiles and urban/rurality categories.[26,27]

Health Data

All admissions to hospital (inclusive of critical care admissions), outpatient, Emergency Department attendances treated in NHS hospitals as well as disease registries and laboratory test results data are available for cohort participants, GP data for diagnoses and treatments from SAIL providing practices are data for approximately 80% of the population.[28]

For peer review only

All relevant health events recorded in clinical data sources will be joined onto the WMC to identify diagnosis of conditions, treatments and various significant heath events that occur across multi-sourced linked heath data per person (Table 2 &Figure 4).

Table 2: Clinical data sources available for the WMC.

Data source	Period covered	Number and percentage of WMC individuals with data
Critical Care Data Set (CCDS)	01-01-2007 – 31-12-2019	79,521 (2.7%)
Welsh Cancer Incidence Surveillance Unit (WCISU)	01-01-2000 – 31-12-2016	328,792 (11.3%)
Welsh Results Reporting Services (WRRS)	01-01-2015 – 10-12-2018	1,540,754 (53.1%)
Emergency Department Data Set (EDDS)	01-04-2009 – 31-12-2019	1,579,665 (54.4%)
Patient Episode Database for Wales (PEDW)	01-01-2000 – 31-12-2019	2,129,384 (73.4%)
Out Patient Dataset for Wales (OPDW)	01-04-2004 – 31-12-2019	2,177,081 (75.0%)
Welsh Longitudinal General Practice (WLGP)	01-01-2000 – 31-12-2019	2,400,313 (82.7%)
Please note clinical data sources will be updated on a monthly/quarterly basis		

The Upset plot in Figure 4 demonstrates the number of WMC participants that have interacted with the various health care settings from 1st January 2000 to their cohort censorship end date.[29] For example, 780,830 (26.9%) individuals have utilised GP, inpatient, outpatient and emergency department services as well as had at least one laboratory test within their WMC coverage.

Phenotyping the e-cohort

Published comorbidity indices and phenotype code lists (International Classification of Diseases 10th revision (ICD-10), OPCS Classification of Interventions and Procedures version 4 (OPCS4) and primary care Read Codes version 2) will be used to measure and conceptualise multi-morbidity. These include those created by: CALIBER initiative; Charlson Comorbidity Index; Common Mental Disorders (CMD); Elixhauser Comorbidity

For peer review only - http://bmjopen.bmj.com/site/about/guidelines.xhtml

Index; Global Burden of Disease Study; and the NHS Quality and Outcomes Framework (QOF).[30-41] Diagnostic codes relating to HIV will not be included in any outputs to conform with SAIL policies. They are part of the list of redacted codes not allowed to be used for research using the data.[42] All ICD-10 and OPCS4 codes provided at the three character level were expanded to include all children terms.

1. **CALIBER**

Phenotyping algorithms created from the CALIBER resource using ICD-10, OPCS4 and Read Codes will be utilised to identify 300 physical and mental health conditions recorded in both primary and secondary healthcare.[31,39]

There are 1,645 distinct ICD-10 codes (at three and four-character level) for 300 conditions, however, when capturing all ICD-10 codes to include variation in coding entry (e.g. C796– instead of C796) and expanding the code list to the four-character level (F200 instead of F20), there are 3,702 distinct ICD-10 codes (at the four-character level) recorded in the inpatient data. This is important to note as to link solely on standardised codes would result in loss of information and potential reporting of false negatives.

There are 587 distinct OPCS4 codes (at three and four-character level) for 28 conditions and 8,588 distinct Read Codes (at the five-character level) for 275 conditions.

2. **Charlson Comorbidity Index**

The Aylin and Bottle Charlson amended ICD-10 code list will be utilised for inpatient diagnosis and the Metcalfe et al (2019) Charlson Read Code list will be utilised for primary care recorded diagnosis.[32,33]

The ICD-10 codes have been taken from the pool of diagnosis codes recorded within hospital admissions data, containing 1,024 distinct codes (at the four-character level) for 16 conditions. The GP data contains 4,545 distinct Read Codes at the five-character level.

3. **Common Mental Disorders (CMD)**

The John et al, 2016 validated algorithm will be used to identify CMD in GP data.[30,40,41] The algorithm has utilised a combination of diagnosis, treatment and symptoms Read Codes in identifying CMD. Individuals with CMD are identified as either having a historical diagnosis code, currently treated or, having a current diagnosis/current symptom code. There are 89 distinct diagnosis codes, 15 symptom codes and 601 treatment codes.

4. **Elixhauser Comorbidity Index**

The Quan et al (2005) Elixhauser ICD-10 code list will be utilised for inpatient diagnosis and the Metcalfe et al (2019) Elixhauser Read Code list will be utilised for primary care recorded diagnosis.[33,34]

For peer review only

The ICD-10 codes have been taken from the pool of diagnosis codes recorded within hospital admissions data and contains 1,404 distinct codes (at the four-character level) for 30 conditions. The General practice data contains 6,074 distinct Read codes at the five-character level.

5. **Global Burden of Disease (GBD) Study**

The GBD 2019 ICD-10 codes will be used to identify 130 health conditions in secondary healthcare data. There are 3,497 distinct ICD-10 codes at the three and four-character level.[38]

6. **Quality Outcome Framework (QOF)**

The QOF conditions business rule V38 will be used to identify 18 health conditions in primary care data.[35] The 18 conditions are asthma, atrial fibrillation, obesity, coronary heart disease, chronic obstructive pulmonary disease, cancer, chronic kidney disease, dementia, depression, diabetes, epilepsy, heart failure, hypertension, learning difficulties, peripheral arterial disease, rheumatoid arthritis, serious mental illness and stroke. There are 2,275 distinct Read Codes available at the five-character level for the 18 QOF conditions.

Statistical analysis

The WMC provides an accessible research ready data asset to further understanding of multi-morbidity through the use of bio-statistical and machine learning approaches. Our collaborative team will work across a number of projects to develop and evaluate statistical and machine learning algorithms to address the following broad analytical challenges:

- What is the prevalence of multi-morbidity in the WMC, and how does prevalence of multi-morbidity change over time?
- What are common clusters of multi-morbidity in the WMC, and how do they correspond to or differ from, common clusters of multi-morbidity identified in other datasets?
- Which clusters of multi-morbidity occur less frequently than one would expect based on the prevalence of their constituent conditions?
- How does multi-morbidity develop across the life course (i.e. trajectories)?
- What are the biological, psychological, and social determinants of different clusters and trajectories of multi-morbidity?
- Which clusters and trajectories of multi-morbidity are associated with poor health outcomes?
- Which clusters and trajectories of multi-morbidity are associated with high service utilisation?
- Does multi-morbidity in specific groups (e.g. patients with musculoskeletal conditions) differ from multi-morbidity in general?

The overarching aim is to evaluate and provide new multi-morbidity phenotypes and algorithms to the NHS and research communities to support prevention, healthcare planning and the management of individuals with multi-morbidity.

For peer review only

We will draw upon both methods from statistics (e.g. regression analysis, longitudinal mixed models, multiple correspondence analysis, factor analysis [43], multi-state models, and latent class analysis) and machine learning (e.g. k-means clustering, semantic similarity clustering, market basket analysis, network models [44], and deep learning). We will use resampling methods to assess the stability of identified multi-morbidity clusters, and develop visualisation techniques to summarise multi-morbidity clusters and their associations with risk factors and outcomes.

Analyses will be coded in R, WinBUGS, and Python and made available to WMC users via a Git library to maximise transparency and reproducibility.[45]

Patient and public involvement

The proposal to develop WMC was submitted to the independent Information Governance Review Panel (IGRP) that includes members of the public (IGRP Project: 0911). We worked with this group to refine the study protocol. The scientific steering group includes two members of the public who have contributed to this paper. The HDR UK National Implementation Project Multi-morbidity Resource has a work package on PPI with a panel drawn from across the UK meeting to discuss the research work and feed into the research and dissemination plans.

Ethics and dissemination

The use of de-identified data in SAIL complies with National Research Ethics Service (NRES) guidance.[46] Applications to use data held within the SAIL Databank, an ISO: 27001 and UKSA DEA accredited TRE, must first be approved by the independent Information Governance Review Panel (IGRP). This panel contains individuals with expertise in data governance and protection, including the Chair of the Wales NRES Committee, Caldicott Guardians, and members of the public. WMC was approved by IGRP on 26th June 2019.

Findings from this study will be disseminated widely through a variety of routes, including to health policy and NHS leads across UK, the Academy of Medical Sciences and the Royal Colleges, as well as traditional scientific outlets. The team includes NHS clinicians and informaticians to allow for early NHS adoption of useful findings. Members of the public embedded in the team will create plain English summaries and lead at public facing meetings.

Contributors

Conceptualisation of study JL, AA, UA, GH, CM, DOR, RAL; data curation and analysis JL; original draft writing JL, review and editing of manuscript JL, AA, UA, GH, AAL, RB, JR, AW, RF, CM, CD, JPR, NP, CH, SD, RO, KRA, AJ, DOR, SR, MH, CPG, JD, CD,LC, JG, JC, AJB, RAL

For peer review only

Acknowledgements

This study makes use of anonymised data held in the Secure Anonymised Information Linkage (SAIL) Databank. We would like to acknowledge all the data providers and people who make anonymised data available for research. The authors would like to extend their gratitude and acknowledgement to the NHS, the SAIL Consumer panel as well as the IGRP who approved this project.

Funding

Two UK-wide collaborative efforts have been formed to address several of the AMS report priorities: *Measuring and Understanding Multi-morbidity using Routine Data in the UK – (MUrMuRUK)* and *Application of machine learning to discover new multi-morbidity phenotypes associated with poorer outcomes (WMML).*

This work was supported by Health Data Research UK (HDR-9006; CFC0110) and the Medical Research Council (MR/S027750/1). Health Data Research UK is funded by: UK Medical Research Council; Engineering and Physical Sciences Research Council; Economic and Social Research Council; National Institute for Health Research (England); Chief Scientist Office of the Scottish Government Health and Social Care Directorates; Health and Social Care Research and Development Division (Welsh Government); Public Health Agency (Northern Ireland); British Heart Foundation; and Wellcome Trust.

Disclaimer

The views and opinions expressed therein are those of the authors and do not necessarily reflect those of the funding agencies, NHS organisations or Welsh Government.

Competing interests

None declared.

Figure captions

Figure 1**:** WMC flow diagram, based on inclusion criteria.

Figure 2: WMC pyramid for age (years) at cohort inception.

Figure 3: Heatmap of person years of WMC follow up, by age group, sex and area level deprivation at cohort inception.

Figure 4: Number of WMC individuals utilising healthcare services recorded in multi-source data sources, 20 most common combinations presented.

For peer review only

References

1. The Academy of Medical Sciences. Multimorbidity: a priority for global health research, April 2018. https://acmedsci.ac.uk/file-download/82222577

2. WHO. World Health Organization. The Challenges of a changing world. The World Health Report 2008—primary Health Care (Now More Than Ever). 2008 https://journals.plos.org/plosone/article/file?id=10.1371/journal.pone.0237186&type=printable

3. Barnett K, Mercer SW, Norbury M, Watt G, Wyke S, Guthrie B. Epidemiology of multimorbidity and implications for health care, research, and medical education: a cross-sectional study. The Lancet. 2012;380(9836):37–43.
4. Cassell A, Edwards D, Harshfield A, Rhodes K, Brimicombe J, Payne R, et al. The epidemiology of multimorbidity in primary care: a retrospective cohort study. British Journal of General Practice. 2018;68(669).
5. Nunes BP, Flores TR, Mielke GI, Thumé E, Facchini LA. Multimorbidity and mortality in older adults: A systematic review and meta-analysis. *Arch Gerontol Geriatr*. 2016;67:130-138. Doi:10.1016/j.archger.2016.07.008
6. Hall, M., Dondo, T. B., Yan, A. T., Mamas, M. A., Timmis, A. D., Deanfield, J. E., ... & Gale, C. P. (2018). Multimorbidity and survival for patients with acute myocardial infarction in England and Wales: Latent class analysis of a nationwide population-based cohort. *PLoS medicine*, *15*(3), e1002501.
7. Kingston A, Robinson L, Booth H, et al. Projections of multi-morbidity in the older population in England to 2035: estimates from the Population Ageing and Care Simulation (PACSim) model. Age Ageing. 2018. doi: 10.1093/ageing/afx201
8. Diederichs C, Berger K, Bartels D. The measurement of multiple chronic diseases—a systematic review on existing multimorbidity indices. J Gerontol A Biol Sci Med Sci 2011; 66: 301–11.
9. Ford JC, Ford JA. Multimorbidity: will it stand the test of time? Age Ageing. 2018;47(1):6–8.
10. Vos T, Flaxman AD, Naghavi M, Lozano R, Michaud C, Ezatti M, et al. Years lived with disability (YLDs) for 1160 sequelae of 289 diseases and injuries 1990—2010: a systematic analysis for the Global Burden of Disease Study 2010. The Lancet 2012;380(9859):2163 – 2196. (15 December 2012). doi:10.1016/S0140-6736(12)61729-2.
11. Steel N, Ford J, Newton J, Davis A, Vos T, Naghavi M et al. Mortality, causes of death, years of life lost, years lived with a disability, and disability-adjusted life years in the countries of the UK and 150 English Local Authority areas 1990-2016: a systematic analysis for the Global Burden of Disease Study 2016. Accepted Lancet 02/09/18.
12. Bauermeister S, Orton C, Thompson S. et al. The Dementias Platform UK (DPUK) Data Portal. European Journal of Epidemiology 2020;35:601-611. https://doi.org/10.1007/s10654-020-00633-4
13. Lyons RA, Jones KH, John G, Brooks CJ, Verplancke JP, Ford DV, Brown G, Leake K. The SAIL databank: linking multiple health and social care datasets. BMC Med Inform Decis Mak. 2009 Jan 16;9:3. http://www.biomedcentral.com/1472-6947/9/3
14. Ford DV, Jones KH, Verplancke JP, Lyons RA, John G, Brown G, Brooks CJ, Thompson S, Bodger O, Couch T, Leake K. The SAIL Databank: building a national architecture for e-health research and evaluation. BMC Health Services Research 2009;9:157 doi:10.1186/1472-6963-9-157
15. Lyons RA**,** Ford DV, Moore L, Rodgers SE. Using data linkage to measure the population health impact of non-healthcare interventions. The Lancet 2014;383:1517-1518.
16. Lyons RA, Turner S, Lyons J, Walters A, Snooks HA, Greenacre J, Humphreys C, Jones SJ. All Wales Injury Surveillance System revised: development of a population based system to evaluate single and multi-level interventions. Inj Prev 2015;0:1-6. Published Online First: [09/12/15] doi:10.1136/injuryprev-2015-041814
17. Snooks HA, Anthony R, Chatters R, Dale J, Fothergill R, Gaze S, et al. Support and Assessment for Fall Emergency Referrals (SAFER) 2: a cluster randomised trial and systematic review of clinical effectiveness and cost-effectiveness of new protocols for emergency ambulance paramedics to

assess older people following a fall with referral to community-based care when appropriate. Health Technology Assessment. 2017;21(13):1–218.
18. Hollingsworth JP, Rodgers SE, Akbari A, Mizen A, Berridge DM, Clegg A, Walters A, Lyons RA, Williams N. A study protocol for investigating the impact of community home modification services on hospital utilisation for fall injuries: a controlled longitudinal study using data linkage. BMJ Open 2018:8:e026290. doi: 10.1136/bmjopen-2018-026290.
19. Mizen A, Song J, Fry R, Akbari A, Berridge D, Johnson R, Rebecca Lovell R, Lyons RA, Mark Nieuwenhuijsen M, Gareth Stratton G, Wheeler BW, White J, White M, Rodgers S. Longitudinal access and exposure to green-blue spaces and individual-level mental health and wellbeing: A study programme protocol for a longitudinal, population-wide record-linked natural experiment. BMJ Open 2019;9:e027289. Doi:10.1136/bmjopen-2018-027289
20. Szakmany S, Walters AM, Pugh R, Battle C, Berridge DM, Lyons RA. Risk factors for 1-year mortality and healthcare utilisation patterns in critical care survivors: a retrospective, observational, population-based data-linkage study. Crit Care Med 2019;47:15-22. doi: 10.1097/CCM.0000000000003424
21. Rodgers SE, Bailey R, Johnson R, Berridge DM, Poortinga W, Lannon S, Smith R, Lyons RA. Emergency hospital admissions associated with a non randomised housing intervention meeting national housing quality standards: a longitudinal data linkage study. J Epidemiol Community Health 2018;0:1-8 doi: 10.1136/jech-2017-210370
22. Paranjothy S, Evans A, Bandyopathy A, Fone D, Schofield B, John A, Bellis MA, Lyons RA, Farewell D, Long SL. Risk of emergency hospital admissions associated with mental disorders and alcohol misuse in the household: an electronic birth cohort study. Lancet Public Health 2018;3;e279-288. https://doi.org/10.1016/S2468-2667(18)30069-0 .
23. Schnier C, Wilkinson T, Akbari A, Orton C, Sleegers K, Gallacher J, Lyons RA, Sudlow CLM, on behalf of Dementias Platform UK. Cohort profile: The Secure Anonymised Information Linkage Databank Dementia e-cohort (SAIL-DeC). IJPDS 2020 (published 25/01/20) https://doi.org/10.23889/ijpds.v5i1.1121.
24. Discontinuities in results for health-related lifestyle and general health between the Welsh Health Survey and National Survey for Wales. Available: https://gov.wales/sites/default/files/statistics-and-research/2019-02/discontinuities-results-health-related-lifestyle-general-health-between-welsh-health-survey-national-survey-wales-2018.pdf [Accessed 24 August 2020].
25. Rodgers SE, Lyons RA, Dsilva R, Jones KH, Brooks CJ, Ford DV, et al. Residential Anonymous Linking Fields (RALFs): a novel information infrastructure to study the interaction between the environment and individuals' health. Journal of Public Health. 2009;31(4):582–8.
26. Welsh Index Multiple Deprivation Index. Available: https://gov.wales/welsh-index-multiple-deprivation-index-guidance [Accessed 9 April 2020].
27. 2011 rural/urban classifications. Available: https://www.ons.gov.uk/methodology/geography/geographicalproducts/ruralurbanclassifications/2011ruralurbanclassification [Accessed 9 April 2020].
28. Thayer D, Rees A, Kennedy J, Collins H, Harris D, Halcox J, et al. Measuring follow-up time in routinely-collected health datasets: Challenges and solutions. Plos One. 2020;15(2).
29. Nils Gehlenborg (2019). UpSetR: A More Scalable Alternative to Venn and Euler Diagrams for Visualizing Intersecting Sets. R package version 1.4.0. https://CRAN.R-project.org/package=UpSetR
30. John, A., McGregor, J., Fone, D. *et al.* Case-finding for common mental disorders of anxiety and depression in primary care: an external validation of routinely collected data. *BMC Med Inform Decis Mak* **16,** 35 (2016). https://doi.org/10.1186/s12911-016-0274-7
31. Kuan V, Denaxas S, Gonzalez-Izquierdo A, Direk K, Bhatti O, Husain S, et al. A chronological map of 308 physical and mental health conditions from 4 million individuals in the English National Health Service. The Lancet Digital Health. 2019;1(2).
32. Bottle A, Aylin P. Comorbidity scores for administrative data benefited from adaptation to local coding and diagnostic practices. Journal of Clinical Epidemiology. 2011;64(12):1426–33.
33. Metcalfe D, Masters J, Delmestri A, Judge A, Perry D, Zogg C, et al. Coding algorithms for defining Charlson and Elixhauser co-morbidities in Read-coded databases. BMC Medical Research Methodology. 2019;19(1).

For peer review only - http://bmjopen.bmj.com/site/about/guidelines.xhtml

34. Quan H, Sundararajan V, Halfon P, Fong A, Burnand B, Luthi J-C, et al. Coding Algorithms for Defining Comorbidities in ICD-9-CM and ICD-10 Administrative Data. Medical Care. 2005;43(11):1130–9.
35. Quality and Outcomes Framework (QOF) business rules v 38 2017-2018 October code release. Available: https://digital.nhs.uk/data-and-information/data-collections-and-data-sets/data-collections/quality-and-outcomes-framework-qof/quality-and-outcome-framework-qof-business-rules/quality-and-outcomes-framework-qof-business-rules-v-38-2017-2018-october-code-release [Accessed 21st August 2019]
36. Charlson ME, Pompei P, Ales KL, MacKenzie CR; A new method of classifying prognostic comorbidity in longitudinal studies: development and validation; J Chron Dis, 1987, 40: 373-383.
37. Elixhauser A, Steiner C, Harris R, Coffey RM; Comorbidity Measures For Use With Administrative Data; Medical Care, 1998, 36:8-27
38. Global Burden of Disease Collaborative Network. Global Burden of Disease Study 2017 (GBD 2017) Causes of Death and Nonfatal Causes Mapped to ICD Codes. Seattle, United States of America: Institute for Health Metrics and Evaluation (IHME), 2018. Available at: http://ghdx.healthdata.org/record/ihme-data/gbd-2017-cause-icd-code-mappings[Accessed 01 June 2020]
39. J Am Med Inform Assoc. 2019 Dec 1;26(12):1545-1559. doi: 10.1093/jamia/ocz105.
40. John A, DelPozo-Banos M, Gunnell D, Dennis M, Scourfield J, Ford DV, et al. Contacts with primary and secondary healthcare prior to suicide: case-control whole-population-based study using person-level linked routine data in Wales, UK, 2000-2017. Br J Psychiatry. 2020;1–8.
41. Ware JE Jr, Gandek B. Overview of the SF-36 Health Survey and the International Quality of Life Assessment (IQOLA) Project. J Clin Epidemiol. 1998;51(11):903–12.
42. Legally unsharable clinical codes - NHS Digital - Citizen Space [Internet]. Citizenspace.com. [cited 2020 Nov 9]. Available from: https://nhs-digital.citizenspace.com/standards-assurance/legally-unsharable-clinical-codes
43. Pages J. Multiple factor analysis by example using R [Internet]. Philadelphia, PA: Chapman & Hall/CRC; 2014. Available from: http://dx.doi.org/10.1201/b17700
44. Marx P, Antal P, Bolgar B, Bagdy G, Deakin B, Juhasz G. Comorbidities in the diseasome are more apparent than real: What Bayesian filtering reveals about the comorbidities of depression. PLoS Comput Biol. 2017 Jun 23;13(6):e1005487.
45. Lunn, D.J., Thomas, A., Best, N., and Spiegelhalter, D. (2000) WinBUGS — a Bayesian modelling framework: concepts, structure, and extensibility. *Statistics and Computing*, **10**:325–337.
46. Jones KH et al.The Secure Anonymous Information Linkage (SAIL) Gateway: a case study describing a remote access system for health-related research and evaluation. Journal of Biomedical Informatics 01/2014; DOI:10.1016/j.jbi.2014.01.003

For peer review only

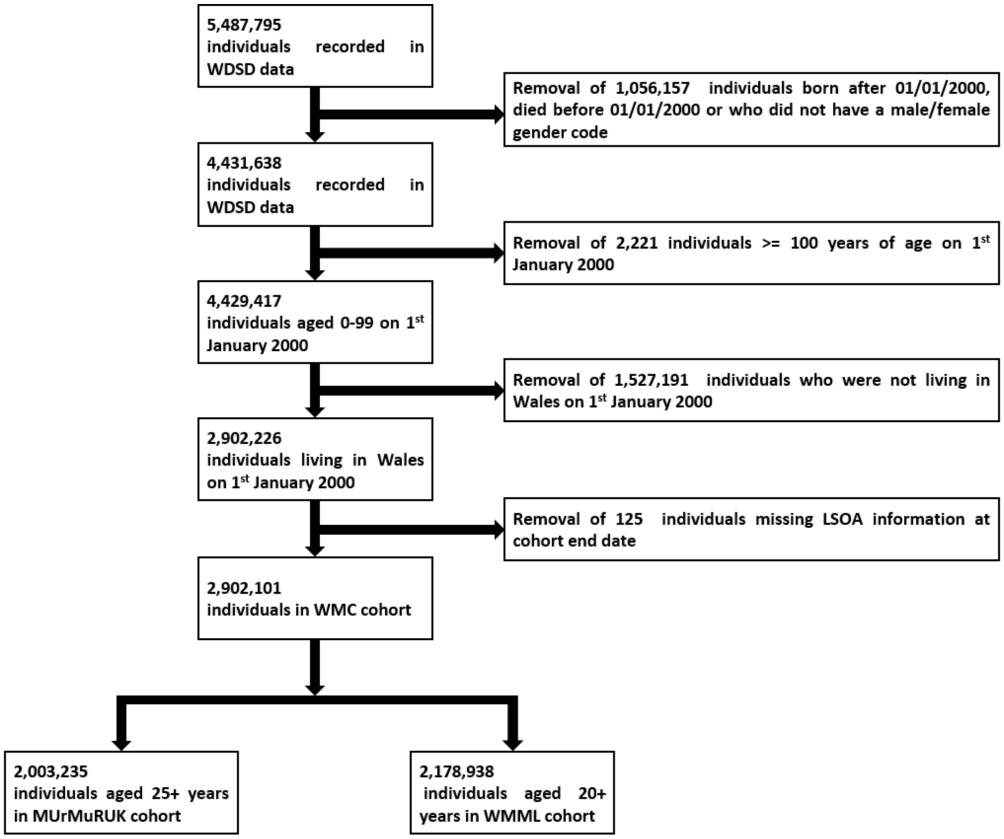

Figure 1: WMC flow diagram, based on inclusion criteria.

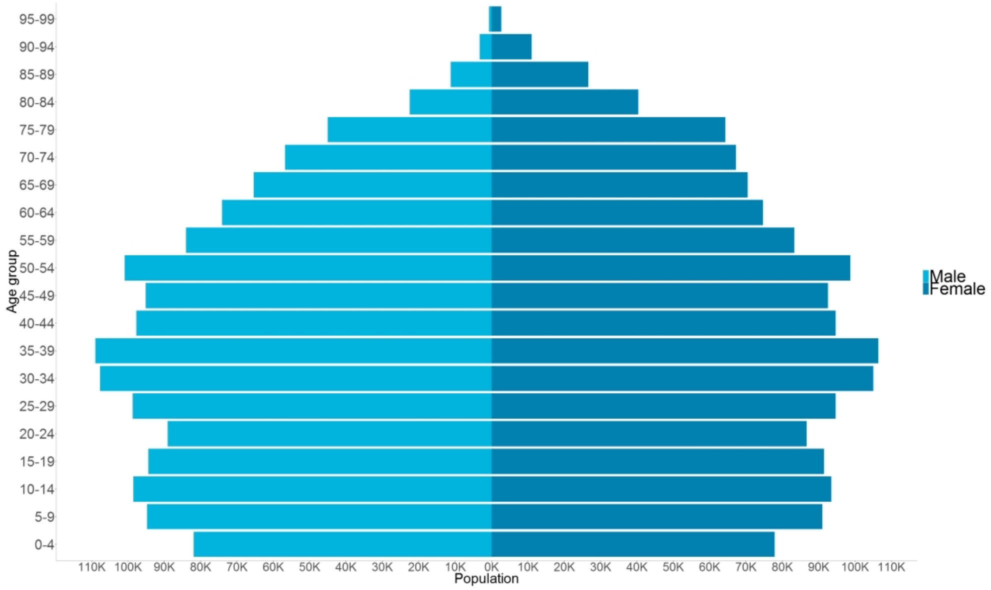

Figure 2: WMC pyramid for age (years) at cohort inception.

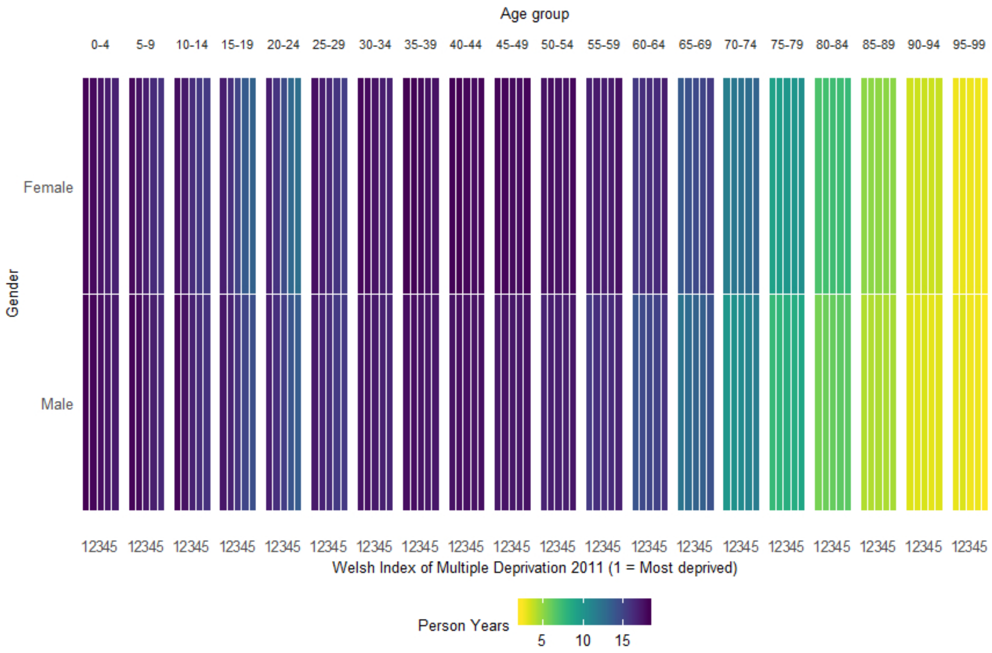

Figure 3: Heatmap of person years of WMC follow up, by age group, sex and area level deprivation at cohort inception.

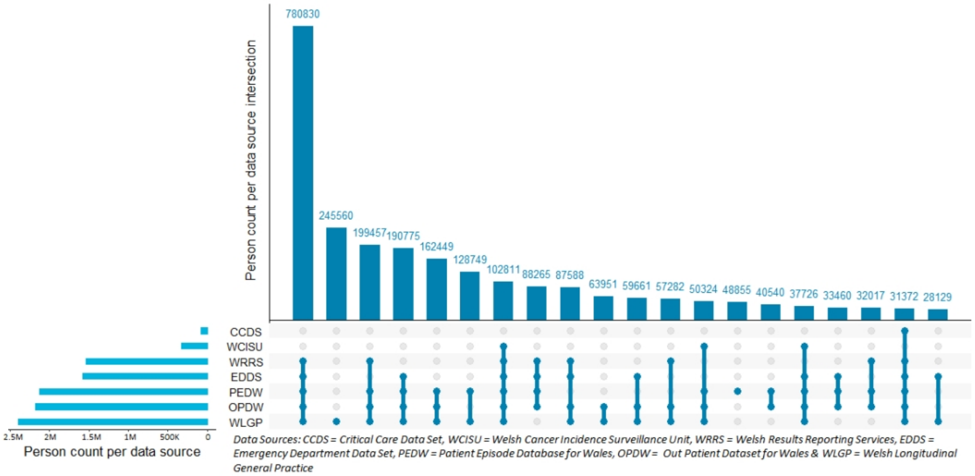

Figure 4: Number of WMC individuals utilising healthcare services recorded in multi-source data sources, 20 most common combinations presented.

Appendix

Table A1: WMC participants categorised by age group and sex at cohort start

Age group	Sex	Count	Percentage
00-04	Male	81,915	2.82
00-04	Female	77,873	2.68
05-09	Male	94,737	3.26
05-09	Female	90,940	3.13
10-14	Male	98,466	3.39
10-14	Female	93,447	3.22
15-19	Male	94,345	3.25
15-19	Female	91,440	3.15
20-24	Male	89,037	3.07
20-24	Female	86,666	2.99
25-29	Male	98,622	3.40
25-29	Female	94,592	3.26
30-34	Male	107,671	3.71
30-34	Female	104,986	3.62
35-39	Male	108,964	3.75
35-39	Female	106,312	3.66
40-44	Male	97,637	3.36
40-44	Female	94,599	3.26
45-49	Male	95,071	3.28
45-49	Female	92,478	3.19
50-54	Male	100,866	3.48
50-54	Female	98,606	3.40
55-59	Male	83,949	2.89
55-59	Female	83,210	2.87
60-64	Male	74,115	2.55
60-64	Female	74,591	2.57
65-69	Male	65,354	2.25
65-69	Female	70,389	2.43
70-74	Male	56,746	1.96
70-74	Female	67,227	2.32
75-79	Male	45,027	1.55
75-79	Female	64,274	2.21
80-84	Male	22,441	0.77
80-84	Female	40,344	1.39
85-89	Male	11,184	0.39
85-89	Female	26,540	0.91
90-94	Male	3,208	0.11
90-94	Female	10,951	0.38
95-99	Male	633	0.02
95-99	Female	2,648	0.09

For peer review only

Table A2: WMC average person years of follow up, categorised by age group, sex and WIMD 2011 at cohort start

Age group	Sex	WIMD 2011 quintiles	Average Pys
00-04	Male	1	18.16
00-04	Male	2	17.93
00-04	Male	3	17.63
00-04	Male	4	17.28
00-04	Male	5	17.11
05-09	Male	1	18.06
05-09	Male	2	17.81
05-09	Male	3	17.26
05-09	Male	4	16.91
05-09	Male	5	16.50
10-14	Male	1	17.93
10-14	Male	2	17.46
10-14	Male	3	16.76
10-14	Male	4	16.24
10-14	Male	5	15.98
15-19	Male	1	17.30
15-19	Male	2	16.73
15-19	Male	3	15.75
15-19	Male	4	14.89
15-19	Male	5	14.34
20-24	Male	1	16.96
20-24	Male	2	16.04
20-24	Male	3	15.29
20-24	Male	4	14.03
20-24	Male	5	13.59
25-29	Male	1	17.18
25-29	Male	2	16.71
25-29	Male	3	16.22
25-29	Male	4	15.57
25-29	Male	5	15.47
30-34	Male	1	17.44
30-34	Male	2	17.41
30-34	Male	3	17.11
30-34	Male	4	16.82
30-34	Male	5	16.60
35-39	Male	1	17.66
35-39	Male	2	17.63
35-39	Male	3	17.41
35-39	Male	4	17.27

For peer review only

35-39	Male	5	17.22
40-44	Male	1	17.50
40-44	Male	2	17.63
40-44	Male	3	17.55
40-44	Male	4	17.40
40-44	Male	5	17.57
45-49	Male	1	17.23
45-49	Male	2	17.39
45-49	Male	3	17.28
45-49	Male	4	17.24
45-49	Male	5	17.48
50-54	Male	1	16.68
50-54	Male	2	16.96
50-54	Male	3	16.89
50-54	Male	4	16.91
50-54	Male	5	17.30
55-59	Male	1	15.46
55-59	Male	2	16.03
55-59	Male	3	16.20
55-59	Male	4	16.31
55-59	Male	5	16.78
60-64	Male	1	14.07
60-64	Male	2	14.64
60-64	Male	3	14.96
60-64	Male	4	15.19
60-64	Male	5	15.80
65-69	Male	1	12.14
65-69	Male	2	12.70
65-69	Male	3	13.24
65-69	Male	4	13.46
65-69	Male	5	14.21
70-74	Male	1	9.73
70-74	Male	2	10.23
70-74	Male	3	10.59
70-74	Male	4	11.02
70-74	Male	5	11.58
75-79	Male	1	7.46
75-79	Male	2	7.73
75-79	Male	3	8.14
75-79	Male	4	8.29
75-79	Male	5	8.79
80-84	Male	1	5.55
80-84	Male	2	5.82
80-84	Male	3	6.02

For peer review only

80-84	Male	4	6.24
80-84	Male	5	6.30
85-89	Male	1	4.20
85-89	Male	2	4.18
85-89	Male	3	4.28
85-89	Male	4	4.27
85-89	Male	5	4.41
90-94	Male	1	3.09
90-94	Male	2	3.07
90-94	Male	3	3.22
90-94	Male	4	2.95
90-94	Male	5	3.13
95-99	Male	1	2.87
95-99	Male	2	3.19
95-99	Male	3	2.64
95-99	Male	4	2.77
95-99	Male	5	2.32
00-04	Female	1	18.03
00-04	Female	2	17.82
00-04	Female	3	17.37
00-04	Female	4	16.99
00-04	Female	5	16.73
05-09	Female	1	17.84
05-09	Female	2	17.44
05-09	Female	3	16.89
05-09	Female	4	16.27
05-09	Female	5	15.92
10-14	Female	1	17.57
10-14	Female	2	17.09
10-14	Female	3	16.20
10-14	Female	4	15.60
10-14	Female	5	15.51
15-19	Female	1	16.93
15-19	Female	2	16.08
15-19	Female	3	14.88
15-19	Female	4	13.68
15-19	Female	5	13.21
20-24	Female	1	16.94
20-24	Female	2	15.81
20-24	Female	3	14.61
20-24	Female	4	12.89
20-24	Female	5	12.48
25-29	Female	1	17.53
25-29	Female	2	16.99

For peer review only

25-29	Female	3	16.47
25-29	Female	4	15.77
25-29	Female	5	15.34
30-34	Female	1	17.94
30-34	Female	2	17.77
30-34	Female	3	17.39
30-34	Female	4	16.94
30-34	Female	5	16.77
35-39	Female	1	18.18
35-39	Female	2	18.12
35-39	Female	3	17.78
35-39	Female	4	17.47
35-39	Female	5	17.49
40-44	Female	1	18.11
40-44	Female	2	18.16
40-44	Female	3	17.91
40-44	Female	4	17.71
40-44	Female	5	17.92
45-49	Female	1	17.92
45-49	Female	2	17.93
45-49	Female	3	17.82
45-49	Female	4	17.66
45-49	Female	5	17.97
50-54	Female	1	17.49
50-54	Female	2	17.69
50-54	Female	3	17.49
50-54	Female	4	17.44
50-54	Female	5	17.87
55-59	Female	1	16.79
55-59	Female	2	17.09
55-59	Female	3	17.00
55-59	Female	4	17.06
55-59	Female	5	17.54
60-64	Female	1	15.53
60-64	Female	2	16.04
60-64	Female	3	16.23
60-64	Female	4	16.28
60-64	Female	5	16.96
65-69	Female	1	13.76
65-69	Female	2	14.28
65-69	Female	3	14.70
65-69	Female	4	14.92
65-69	Female	5	15.52
70-74	Female	1	11.43

For peer review only

70-74	Female	2	11.93
70-74	Female	3	12.27
70-74	Female	4	12.57
70-74	Female	5	13.12
75-79	Female	1	9.06
75-79	Female	2	9.35
75-79	Female	3	9.70
75-79	Female	4	9.82
75-79	Female	5	10.25
80-84	Female	1	6.78
80-84	Female	2	7.07
80-84	Female	3	7.19
80-84	Female	4	7.26
80-84	Female	5	7.50
85-89	Female	1	4.98
85-89	Female	2	5.02
85-89	Female	3	4.97
85-89	Female	4	5.07
85-89	Female	5	5.07
90-94	Female	1	3.45
90-94	Female	2	3.53
90-94	Female	3	3.61
90-94	Female	4	3.55
90-94	Female	5	3.66
95-99	Female	1	2.66
95-99	Female	2	2.87
95-99	Female	3	2.70
95-99	Female	4	2.75
95-99	Female	5	2.48

For peer review only
