# Supplementary material for: Protocol for the development of the Wales Multimorbidity e-Cohort (WMC): data sources and methods to construct a population-based research platform to investigate multimorbidity
Source: BMJ Open. 2021 Jan 19;11(1):e047101. doi: 10.1136/bmjopen-2020-047101 (PMC7817800; doi:10.1136/bmjopen-2020-047101)
Supplement: Reviewer comments [file bmjopen-2020-047101.reviewer_comments.pdf]

PEER REVIEW HISTORY

BMJ Open publishes all reviews undertaken for accepted manuscripts. Reviewers are asked to complete a checklist review form (**http://bmjopen.bmj.com/site/about/resources/checklist.pdf**) and are provided with free text boxes to elaborate on their assessment. These free text comments are reproduced below.

ARTICLE DETAILS

**TITLE (PROVISIONAL)**	Protocol for the development of the Wales Multi-morbidity e-Cohort (WMC): data sources and methods to construct a population-based research platform to investigate multi-morbidity.
**AUTHORS**	Lyons, Jane; Akbari, Ashley; Agrawal, Utkarsh; Harper, Gill; Azcoaga-Lorenzo, Amaya; Bailey, Rowena; Rafferty, James; Watkins, Alan; Fry, Richard; McCowan, Colin; Dezateux, Carol; Robson, John; Peek, N; Holmes, Chris; Denaxas, S; Owen, R; Abrams, Keith; John, Ann; OReilly, Dermot; Richardson, Sylvia; Hall, Marlous; Gale, Chris; Davies, Jan; Davies, Chris; Cross, Lynsey; Gallacher, John; Chess, James; Brookes, Anthony; Lyons, Ronan

This article was not externally reviewed at BMJ Open. Protocols that have been independently assessed prior to submission to BMJ Open are usually fast-tracked to publication on the grounds that further substantial changes will not be possible. This independent assessment will usually be external, independent review for both a substantial grant award from a non-commercial or government funder and ethics approval.
